# Artificial intelligence in the task of segmentation and classification of brain metastases images: current challenges and future opportunities

**DOI:** 10.3389/fneur.2025.1581422

**Published:** 2025-09-23

**Authors:** Yiheng Hu, Chao Gao, Yiren Wang, Zhongjian Wen, Cheng Yang, Hairui Deng, Shouying Chen, Yunfei Li, Haowen Pang, Ping Zhou, Bin Liao, Yan Luo

**Affiliations:** ^1^Department of Medical Imaging, Southwest Medical University, Luzhou, China; ^2^Department of Oncology, The Affiliated Hospital of Southwest Medical University, Luzhou, China; ^3^School of Nursing, Southwest Medical University, Luzhou, China; ^4^Wound Healing Basic Research and Clinical Applications Key Laboratory of Luzhou, School of Nursing, Southwest Medical University, Luzhou, China; ^5^School of Clinical Medicine, Southwest Medical University, Luzhou, China; ^6^Department of Radiology, The Affiliated Hospital of Southwest Medical University, Luzhou, China; ^7^Department of Operating Room, The Affiliated Hospital of Southwest Medical University, Luzhou, China

**Keywords:** brain metastases, artificial intelligence, deep learning, machine learning, radiotherapy, diagnostic imaging

## Abstract

Brain metastases (BM) are common complications of advanced cancer, posing significant diagnostic and therapeutic challenges for clinicians. Therefore, the ability to accurately detect, segment, and classify brain metastases is crucial. This review focuses on the application of artificial intelligence (AI) in brain metastasis imaging analysis, including classical machine learning and deep learning techniques. It also discusses the role of AI in brain metastasis detection and segmentation, the differential diagnosis of brain metastases from primary brain tumors such as glioblastoma, the identification of the source of brain metastases, and the differentiation between radiation necrosis and recurrent tumors after radiotherapy. Additionally, the advantages and limitations of various AI methods are discussed, with a focus on recent advancements and future research directions. AI-driven imaging analysis holds promise for improving the accuracy and efficiency of brain metastasis diagnosis, thereby enhancing treatment plans and patient prognosis.

## 1 Introduction

Brain metastases (BM) are one of the common and severe complications of advanced malignant tumors, often indicating poor prognosis and posing a major challenge to clinical tumor treatment (1). The incidence of brain metastases is high, with estimates suggesting that up to one-third of cancer patients will develop brain metastases (2). With the aging of the global population, advances in systemic treatment, and the widespread use of imaging technologies such as magnetic resonance imaging (MRI), the detection and diagnosis rates of BM have been increasing (3, 4). Among the primary tumors that lead to BM, lung cancer, breast cancer, and melanoma are the most common. However, cases of brain metastasis from gastrointestinal tumors, renal cell carcinoma, and gynecological cancers are also on the rise (4, 5). Moreover, it is worth noting that brain metastases significantly impact patient prognosis regardless of the primary tumor type. Taking breast cancer as an example, patients may survive for an average of up to 28 years, but once brain metastasis occurs, the average survival time is reduced to about 10 months (6). Despite the application of treatments such as monoclonal antibodies, tyrosine kinase inhibitors (TKI), and antibody-drug conjugates (ADC), which have improved overall survival (OS) to some extent, brain metastasis remains a severe challenge in breast cancer treatment. Therefore, there is an urgent need to develop timely and precise imaging detection, segmentation, and classification technologies to detect and diagnose brain metastases early, thus providing patients with more time for treatment and improving prognosis.

First, precise detection and segmentation helps doctors accurately assess tumor size, location, and number, providing accurate targeting for local treatments such as surgery and radiotherapy. Achieving this precise assessment relies on imaging technology support. MRI can detect small lesions with high sensitivity and clearly display critical tumor characteristics, making it the recognized main tool for diagnosing BMs (4, 7, 8). However, computed tomography (CT) scanning still maintains irreplaceable value in preliminary screening of brain metastases, rapid assessment in emergency situations, and special clinical scenarios such as medical institutions with limited equipment conditions or patients with MRI contraindications (9, 10). Based on these imaging technologies, precise treatment of brain metastases can be effectively implemented. Stereotactic radiosurgery (SRS) has become an important treatment modality for brain metastases due to its ability to deliver highly focused radiation to metastatic regions while minimizing damage to surrounding normal brain tissue. The implementation of such precise treatments demands extremely high accuracy in imaging detection and segmentation (11). Nevertheless, traditional manual detection and segmentation methods have obvious limitations. On one hand, manual detection and segmentation processes are time-consuming and cumbersome (12); on the other hand, target delineation is susceptible to observer subjectivity, and differences between physicians may increase uncertainty in radiotherapy planning (2). Therefore, the demand for artificial intelligence-based automated detection and segmentation technologies is increasingly urgent. Currently, many deep learning algorithms have been applied to the detection and segmentation tasks of brain metastasis images (13), aiming to improve management efficiency and treatment outcomes for patients with multiple metastases. Precise automated detection and segmentation not only enhance the reliability and efficiency of treatment planning but also establish an important foundation for subsequent fine-grained analysis based on imaging features and the development of personalized treatment strategies.

The classification of brain metastases faces several challenges, such as nature determination, source identification, and treatment response evaluation. The primary challenge is distinguishing brain metastases from primary brain tumors, such as glioblastoma (GBM). For patients previously diagnosed with malignant tumors, new brain lesions require clarification on whether they are primary brain tumors such as GBM or brain metastases from the primary cancer. GBM and brain metastases demonstrate high radiological similarity, typically presenting as rim-enhancing lesions with surrounding T2 hyperintensity (14), but their treatment strategies differ drastically, making accurate preoperative differentiation crucial. For patients presenting with brain lesions as their primary manifestation, identifying the origin of brain metastases has significant clinical implications. Brain metastases from different primary sites require distinct therapeutic approaches; differentiating between metastases originating from lung cancer, breast cancer, melanoma, and other sources helps guide the selection of personalized treatment protocols such as targeted therapies for specific gene mutations and immune checkpoint inhibitors (15). However, accurately determining the origin of brain metastases poses numerous challenges when definitive information about the primary lesion is lacking. While conventional neuropathological examination serves as the gold standard, it carries surgical risks as an invasive procedure, including hemorrhage, infection, and neurological function impairment. Some patients cannot tolerate such examinations due to poor physical condition or deep-seated lesion location. Additionally, routine radiological assessment, though non-invasive, makes it difficult to accurately determine the origin of brain metastases based solely on manual analysis. In clinical practice, 2–14% of patients present with brain metastases as the initial manifestation without an identified primary tumor (16). For some patients, the primary lesion remains unidentified even until the terminal stage of disease. Failure to promptly and accurately identify the origin leads to difficulties in treatment selection, missing the optimal therapeutic window, and severely affecting patient prognosis. Therefore, developing rapid, reliable, and non-invasive methods for primary tumor identification based on medical imaging, using artificial intelligence technology to assist image analysis and reduce dependence on invasive examinations, serves as an important complementary tool for clinical diagnosis, with significant value for optimizing clinical decision-making processes and improving patient treatment experiences. Beyond determining the primary tumor type, patients with brain metastases who have undergone radiotherapy require differentiation between post-radiation radiation necrosis and recurrent tumors. Research indicates that distinguishing between these two conditions using only MRI is often challenging (17). Although differentiation can be achieved through biopsy, stereotactic biopsy may lead to sampling bias in lesions containing both tumor recurrence and radiation necrosis, and biopsy carries procedure-related risks and cannot be regularly repeated (18). More importantly, the treatment strategies for these two conditions are fundamentally different—tumor recurrence requires continued treatment, while radiation necrosis necessitates cessation of radiotherapy and management of necrotic lesions, making accurate differentiation decisive for treatment decisions. These differential diagnostic tasks—whether distinguishing brain metastases from primary brain tumors, tracing the origin of primary tumors, or differentiating post-radiation necrosis from recurrent tumors—all face challenges of overlapping radiological features and diagnostic difficulties. Therefore, there is an urgent need to leverage artificial intelligence technology to analyze big data from medical imaging, improve diagnostic accuracy and efficiency, and provide stronger support for clinical decision-making.

**Figure 1:**
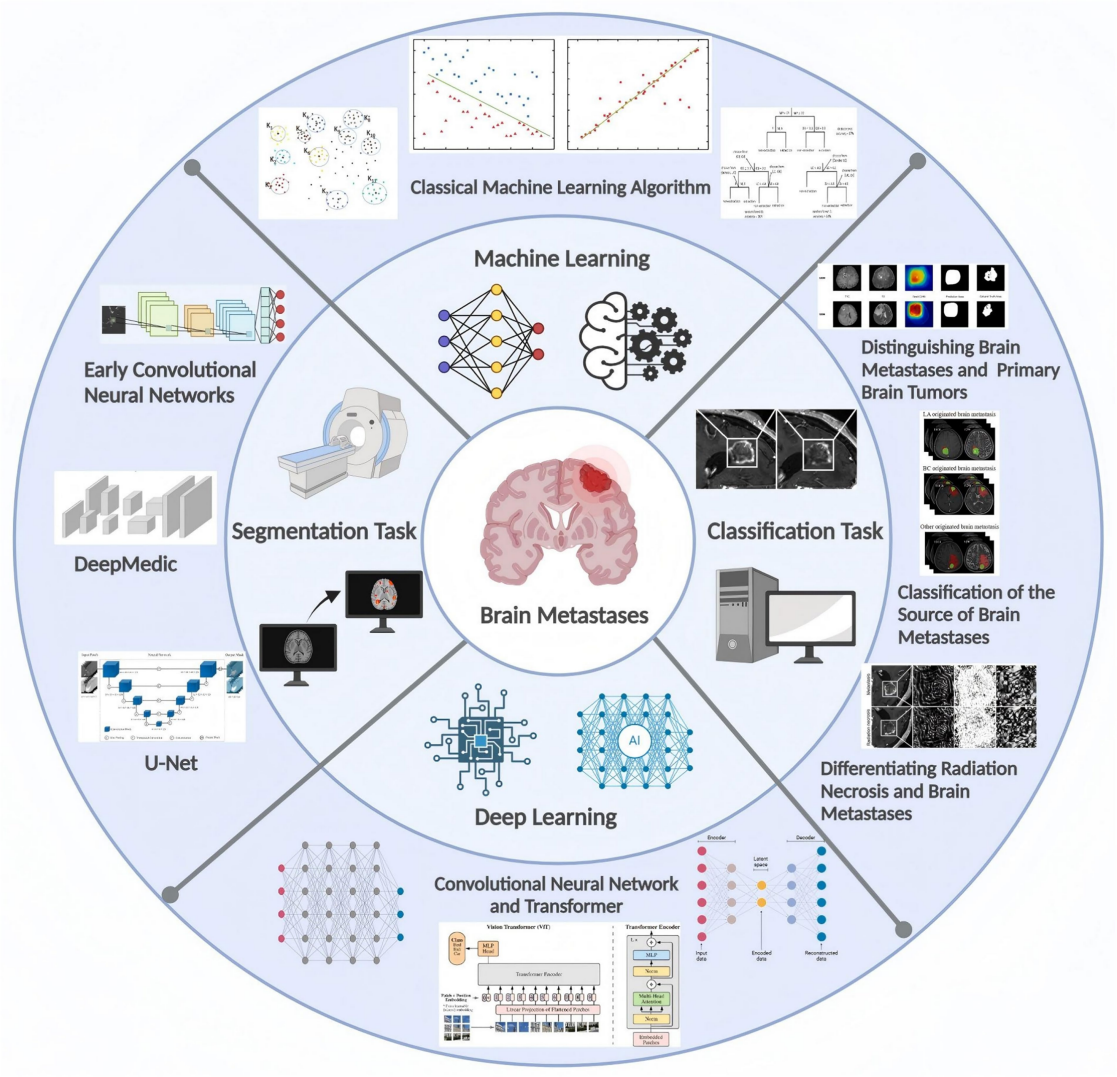
Overview of the main aspects of this review. Referenced and reproduced with permission from Becker et al. (26), Fang et al. (130), Liang et al. (95), Kumar et al. (131), Prasad et al. (129), Hu et al. (46), Park et al. (66), Shi et al. (75), and Larroza et al. (85).

Through early precise imaging analysis and subsequent development of personalized treatment strategies, there is potential to maximize control of tumor progression and improve patient prognosis (19). Therefore, this review focuses on recent advances in artificial intelligence (AI) applications for brain metastasis imaging analysis, encompassing deep learning-based lesion detection and segmentation, differential diagnosis between brain metastases and primary brain tumors, primary tumor origin identification, and differentiation between post-radiation radiation necrosis and recurrent tumors. This paper systematically elucidates the applications and advantages/limitations of classical machine learning methodologies and deep learning algorithms across various tasks, while also projecting future research directions in this field. The aim is to provide more effective decision support for clinical practice and promote further development of relevant artificial intelligence technologies (Figure 1).

## 2 Brain metastases image detection and segmentation

In the field of artificial intelligence, tumor image detection and segmentation represent two core tasks in medical image analysis. Detection tasks aim to identify and localize tumor lesions within images, typically outputting spatial position and bounding box information of the lesions. Segmentation tasks further perform pixel-level precise delineation of tumor regions, assigning category labels to each pixel in the image, thereby achieving accurate tumor contour delineation and regional quantification. These two technologies provide clinicians with critical information such as precise tumor localization, morphological feature analysis, and volumetric measurements, playing important roles in diagnosis, treatment planning, and therapeutic efficacy assessment.

However, the detection and segmentation of brain metastases present unique clinical challenges. Brain metastases often manifest as multiple small lesions, with individual lesions potentially measuring only a few millimeters in diameter and exhibiting relatively low contrast with surrounding brain tissue on MRI images (20). These microscopic lesion characteristics make radiologists prone to missing them during visual identification, particularly when images contain noise and artifacts that further compromise accurate assessment. The scattered distribution and uncertain number of lesions additionally increase the workload and risk of missed diagnoses in manual detection (20–22). Meanwhile, the low contrast features also make it difficult for physicians to precisely delineate lesion boundaries, affecting the accuracy of subsequent treatment planning (20).

Under these specific clinical circumstances, although classical machine learning methods such as threshold segmentation (23) and region growing algorithms (24) possess advantages including strong interpretability, low computational cost, and minimal hardware requirements (25–27), they demonstrate significant technical limitations when confronting the complex characteristics unique to brain metastases, including small lesions, low contrast, and multifocal distribution. These methods are relatively sensitive to image quality and noise, and struggle to effectively capture the diversity of tumor morphology and irregularity of boundaries (28, 29), thereby limiting their widespread application in brain metastases detection and segmentation.

To address these challenges, researchers have developed various deep learning-based detection and segmentation methods, with technological evolution progressing from early CNN local feature extraction, to U-Net’s global–local information fusion, then to DeepMedic’s specialized 3D processing, and more recently to the intelligent development of adaptive frameworks. Deep learning technology, leveraging its powerful feature learning capabilities and effective utilization of large-scale data, has demonstrated significant advantages in processing complex image features and achieving high-precision segmentation, gradually becoming the mainstream technology in this field. In light of this, this review will focus on the applications of deep learning networks and their variants in brain metastases image detection and segmentation.

### 2.1 Early convolutional neural networks and variants

**Figure 2:**
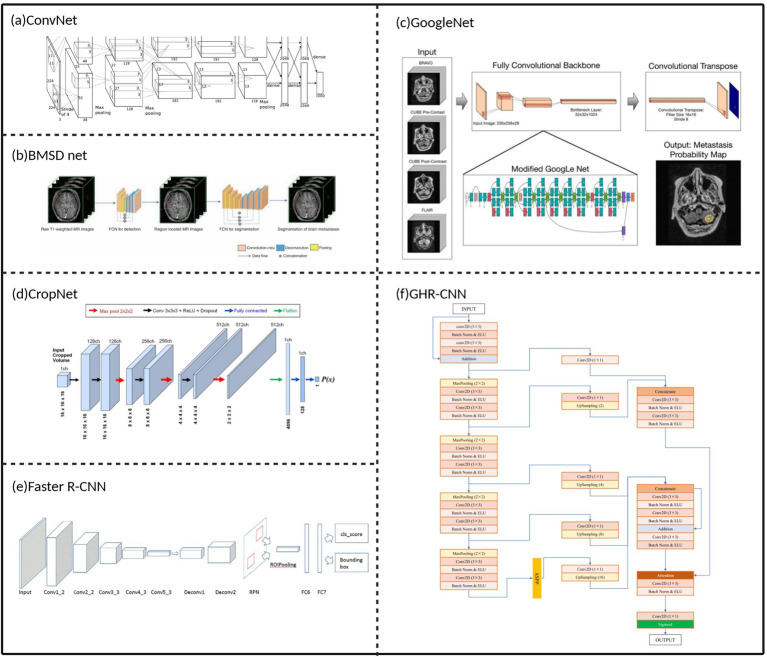
Early convolutional neural network architectures for image segmentation of brain metastases. **(a)** Typical architecture of a ConvNet. **(b)** Network architecture of BMDS net. **(c)** The modified GoogLeNet architecture. **(d)** Network architecture of CropNet. **(e)** Structure of our deep-learning approach faster R-CNN. **(f)** Network architecture of a gated high-resolution neural network. Referenced and reproduced with permission from Losch (31), Xue et al. (32), Grøvik et al. (12), Dikici et al. (33), Zhang et al. (122), and Qu et al. (34).

Convolutional neural networks (CNNs) were among the earliest deep learning networks applied to brain metastases image detection and segmentation (Figure 2). CNNs extract local features from images through convolutional layers and utilize pooling layers to reduce computational load while increasing feature robustness (30). Losch et al. (31) pioneered the application of ConvNet to brain metastases segmentation in 2015, achieving 82.8% sensitivity in detecting lesions larger than 3 millimeters, laying the foundation for deep learning applications in this field. However, early CNN methods exposed significant technical limitations, including high false positive rates (false positive rate of 0.05 per slice), insufficient segmentation accuracy for small metastatic lesions, and deficiencies in feature extraction, multi-scale information fusion, and contextual information utilization. The fundamental cause of these problems lies in the fact that traditional CNN feedforward structures lack global contextual modeling capabilities, relying solely on local convolutions making it difficult to accurately distinguish subtle differences between lesions and normal brain tissue.

To overcome the limitations of traditional CNN architectures, researchers have pursued technical improvements from different perspectives. In terms of dimensional extension, the 2.5D GoogLeNet CNN model proposed by Grøvik et al. (12) attempted to strike a balance between computational efficiency and feature capture capability, better capturing inter-slice features while avoiding the computational burden of full 3D CNNs. However, this method still exhibited performance limitations in false positive control and multiple lesion detection. Regarding network architecture design, the BMDS Net cascaded 3D fully convolutional network proposed by Xue et al. (32) adopted a two-stage strategy of detection-localization followed by segmentation, improving segmentation accuracy to some extent while reducing computational complexity. Nevertheless, it still faced challenges when handling tasks involving discrimination of adjacent lesions or small-volume lesions. These early improvement efforts demonstrated that simply increasing architectural complexity cannot fundamentally resolve the core problems of CNNs in brain metastases analysis.

Recognizing the significant impact of detection tasks on segmentation performance, researchers began exploring CNN methods specifically optimized for brain metastases detection. The CropNet proposed by Dikici et al. (33) focused on the detection task of small brain metastases (≤15 mm), achieving accuracy levels comparable to large lesion detection methods for small lesions through sensitivity-constrained LoG candidate selection and targeted data augmentation strategies. The important significance of this work lies in demonstrating that precise lesion localization can effectively assist subsequent segmentation tasks. Building on this understanding, Qu et al. (34) further proposed the gated high-resolution CNN (GHR-CNN), which achieved improvements in segmentation accuracy, sensitivity, and generalization capability by maintaining high-resolution features and introducing gating mechanisms, particularly excelling in small lesion detection. This indicates that through carefully designed network structures and training strategies, a single segmentation network can also achieve good performance without strictly relying on independent detection steps.

Although CNN methods have made preliminary progress in brain metastases detection and segmentation tasks (Table 1), their inherent technical limitations restrict further performance improvements. Future CNN improvement directions should focus on collaborative optimization of detection and segmentation tasks as well as targeted network structure design. For example, architectures that fuse object detection with instance segmentation, such as Mask R-CNN, provide new technical approaches. However, the key lies in how to effectively integrate detection information into the segmentation process and how to design specialized network structures and training strategies tailored to the specific characteristics of brain metastases.

**Table 1:** CNN-based architecture for brain metastasis segmentation.

Author (year)	Dataset size and source	Imaging modality	Methodology	Model validation methods	Clinical outcomes predicted	Evaluation metrics
Losch (2015) (31)	490 patients, single-center study	3D MRI (T1c)	Multi-scale ConvNet	Internal validation	Segmentation	Sensitivity: 82.8% AFP: 7.7
Grøvik et al. (2020) (12)	156 patients, single-center study	2.5D MRI (T1, T1c, T2, FLAIR)	GoogLeNet	Internal validation	Segmentation	AUC: 0.98 ± 0.04 Precision: 0.79 ± 0.20 Recall: 0.53 ± 0.22 Dice score: 0.79 ± 0.12
Xue et al. (2020) (32)	1,652 patients, multicenter study	3D MRI (T1)	BMDS net	Internal and external validation	Detection and segmentation	Recall: 0.96 ± 0.03 Specificity: 0.99 ± 0.0002 Dice score: 0.85 ± 0.08
Noguchi et al. (2020) (121)	444 patients, single-center study	2D MRI (T1c)	AlexNet, GoogLeNet	Internal validation	Detection	AlexNet Accuracy: 50% Recall: 28% Specificity: 95%
						GoogLeNet: Accuracy: 45% Recall: 27% Specificity: 83%
Dikici et al. (2020) (33)	158 patients, single-center study	3D MRI (T1c)	CropNet	Internal validation	Detection	AFP: 9.12 Sensitivity: 90%
Zhang et al. (2020) (122)	121 patients, single-center study	3D MRI (T1c)	Faster R-CNN	Internal validation	Detection	AUC: 0.79 Recall: 87.1%
Kottlors et al. (2021) (123)	85 patients, single-center study	2D MRI (T1c, BB)	CNN	Internal validation	Detection	Accuracy: 85.5% AUC: 0.87
Qu et al. (2023) (34)	1,592 patients, multicenter study	3D MRI (T1c)	GHR-CNN	Internal and external validation	Detection and segmentation	Recall: 85% Dice score: 0.89 PPV: 93% AFP: 1.07

### 2.2 U-Net and its variants

**Figure 3:**
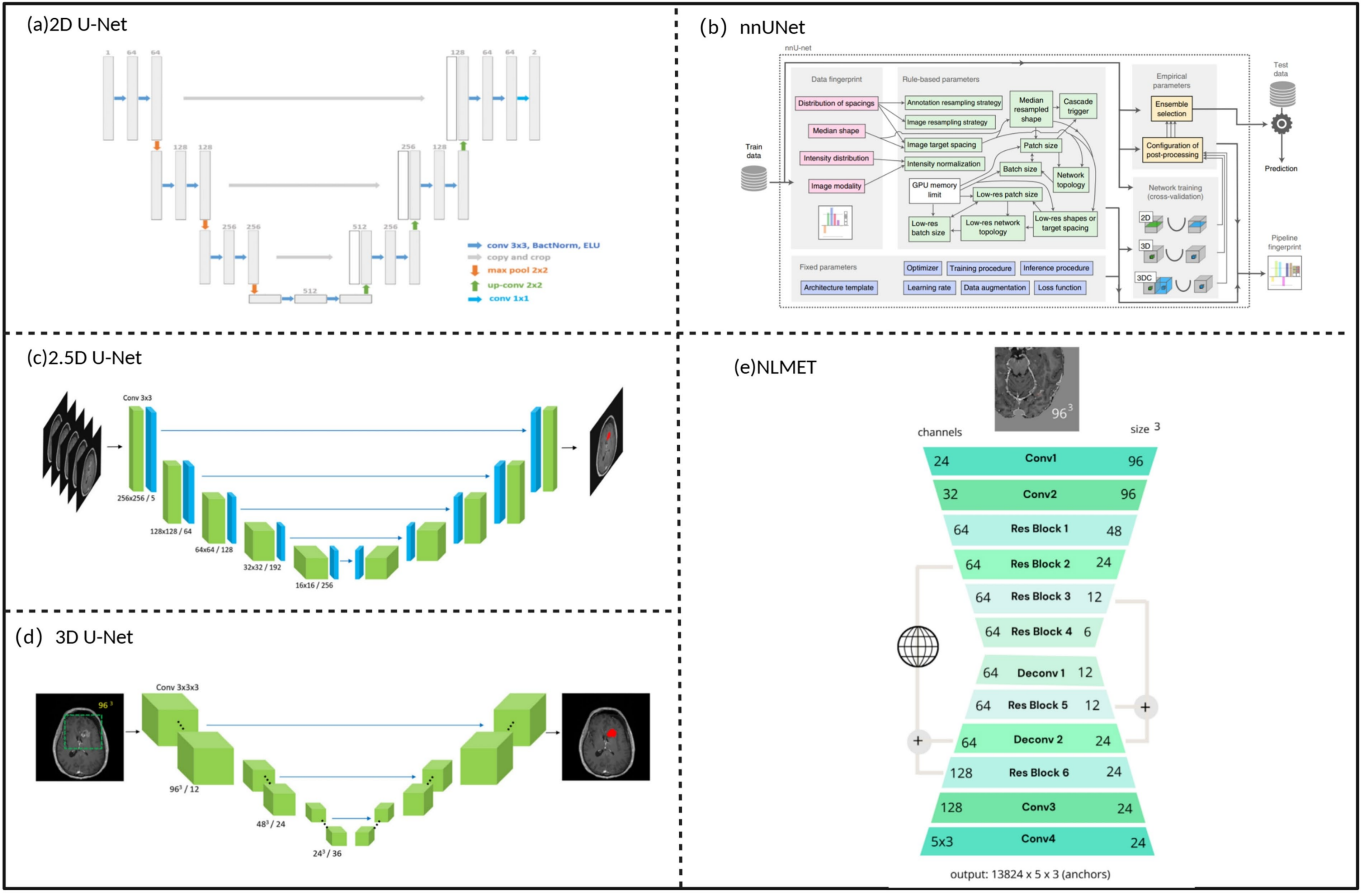
U-Net architecture for image segmentation of brain metastases. **(a)** Typical architecture of a 2D U-Net. **(b)** Network architecture of nnU-Net. **(c)** Typical architecture of a 2.5D U-Net. **(d)** Typical architecture of a 3D U-Net. **(e)**, Structure of NLMET. Referenced and reproduced with permission from Yoo et al. (100), Pflüger et al. (39), Yoo et al. (37), and Liew et al. (38).

The limitations exposed by CNN methods in brain metastases analysis prompted researchers to seek more advanced network architectures. The U-Net architecture, through its encoder-decoder structure and skip connection design, can effectively address the deficiencies of CNNs in capturing global contextual information (Figure 3). The U-Net architecture proposed by Ronneberger et al. (35) in 2015, with its symmetric network design, enables the model to capture both high-level semantic information and preserve low-level detailed features, thus demonstrating good performance in both detection and segmentation tasks of brain metastases.

In the early stages of U-Net application to brain metastases analysis, researchers primarily enhanced the model’s feature extraction capabilities in detection and segmentation tasks through multimodal MRI data fusion. Bousabarah et al. (20) proposed an ensemble learning method based on multimodal 3D MRI data, combining three network structures: cU-Net, moU-Net, and sU-Net, trained with multimodal data including T1c, T2, T2c, and FLAIR, achieving good results in detecting larger volume lesions (>0.06 mL). However, multimodal fusion strategies still exhibited performance limitations in small lesion detection. Addressing this issue, Cao et al. (21) proposed an asymmetric UNet architecture (asym-UNet) from an architectural design perspective, employing different-sized convolutional kernels (3 × 3 × 3 and 1 × 1 × 3) to simultaneously process image features of small tumors and boundary information of large metastases, achieving improved results in small lesion detection tasks (diameter <10 mm). This work demonstrated that targeted architectural modifications can more effectively address specific technical challenges compared to simple data fusion.

With the development of 3D medical image processing technology, researchers began exploring more refined optimization strategies to enhance U-Net performance in brain metastases detection and segmentation tasks. Rudie et al. (22) systematically evaluated the segmentation performance of 3D U-Net in large-scale patient samples, providing benchmark data for the clinical application of this architecture. Building on these foundational works, Chartrand et al. (36) improved detection sensitivity for small brain metastases (2.5–6 mm) to 90.9% by introducing volume-aware loss functions, reducing false negative rates compared to traditional CNN models in this size range. The comparative study by Yoo et al. (37) quantified the performance differences between 2.5D and 3D architectures in detection tasks: 3D U-Net demonstrated higher sensitivity in small metastases detection, while 2.5D U-Net achieved higher detection precision. To achieve balance among different performance metrics in detection, researchers proposed weak learner fusion methods for 2.5D and 3D network prediction features, which could reduce false positive predictions for smaller lesions. The 3D non-local convolutional neural network (NLMET) method by Liew et al. (38) pushed the technical boundary of small lesion detection to 1 mm and maintained good generalization performance across different datasets and MRI sequences.

In recent years, the application of adaptive deep learning frameworks such as nnU-Net in brain metastases detection and segmentation tasks marks a new stage in the technological development of this field. Unlike traditional fixed architectural designs, these frameworks can automatically adjust network structures and training parameters according to dataset characteristics. Pflüger et al. (39) applied nnU-Net to brain metastases detection tasks, achieving detection of contrast-enhancing tumors and non-enhancing FLAIR signal abnormal regions without manual adjustment of volume threshold parameters. In their 2025 research work, Yoo et al. (13) achieved 0.904 sensitivity in brain metastases detection tasks while maintaining low false positive rates (0.65 ± 1.17) by introducing tumor volume-adaptive 3D patch adaptive data sampling (ADS) and adaptive Dice loss (ADL). These results indicate that adaptive frameworks capable of automatically adjusting according to data characteristics have performance advantages over manually designed fixed architectures.

Although U-Net-based brain metastases detection and segmentation technologies have achieved substantial progress (Table 2), further improvement in small lesion detection accuracy and effective integration of emerging network architectures remain the main technical challenges currently faced. In small lesion detection optimization, future research can explore targeted loss function designs, such as focal loss (40) and OHEM (41) methods that can effectively handle class imbalance problems and improve detection sensitivity for small lesions. In feature extraction and fusion strategies, multi-scale feature extraction, attention mechanisms, and Transformer-based fusion methods are expected to further improve small lesion recognition capabilities. Additionally, improvement of evaluation metrics is also of significant importance; for example, similarity distance (SimD) (42) can not only consider position and shape similarity but also automatically adapt to evaluation requirements for different-sized objects in different datasets. In network architecture innovation, the successful performance of emerging architectures like Transformers in natural language processing and computer vision fields has drawn considerable attention to their application potential in brain metastases analysis. For example, the nnU-NetFormer (43) method, which integrates transformer modules into the deep structure of the nnU-Net framework, can effectively extract local and global features of lesion regions in multimodal MR images, although current performance validation of such networks mainly focuses on brain tumor image segmentation tasks. Meanwhile, new training strategies such as self-supervised learning and semi-supervised learning may also provide new solutions for improving model performance and data utilization efficiency, aiming to enhance model generalization capability and clinical applicability while maintaining high accuracy.

**Table 2:** U-Net based architecture for brain metastasis segmentation.

Author (year)	Dataset size and source	Imaging modality	Methodology	Model validation methods	Clinical outcomes predicted	Evaluation metrics
Bousabarah et al. (2020) (20)	509 patients, single-center study	3D MRI (T1c, T2, T2c, FLAIR)	cU-Net, moU-Net, sU-Net	Internal validation	Segmentation	Recall: 0.82 Precision: 0.83 Dice score: 0.74
Cao et al. (2021) (21)	195 patients, single-center study	3D MRI (T1c)	asym-UNet	Internal validation	Segmentation	Dice score: 0.84 False positive: 0.24
Rudie et al. (2021) (22)	413 patients, single-center study	3D MRI (T1, T1 c)	3D U-Net	Internal validation	Segmentation	Dice score: 0.75 Recall: 70.0%
Yoo et al. (2021) (37)	442 patients, single-center study	3D MRI (T1c)	2.5D U-Net, 3D U-Net, weak learner fusion, 3D FCOS	Internal validation	Detection and segmentation	Recall: 74% False positive/scan: 0.53 Precision: 75%
Nomura et al. (2021) (94)	470 patients, single-center study	CT, 3D MRI (T1c)	3D U-Net	Internal validation	Segmentation	Dice score: 0.727 ± 0.115
Cho et al. (2021) (124)	194 patients, multicenter study	3D MRI (T1c)	3D U-Net, 2D U-Net	Internal and external validation	Detection and segmentation	1. Time test set 1 Recall: 75.1%. Dice score: 0.69 ± 0.22 2. Geography test set Recall: 87.7% Dice score: 0.68 ± 0.20 Dice score: 0.68 ± 0.20 3. Time test set 2 Recall: 94.7% Dice score: 0.82 ± 0.20 3. Time test set 2 Dice score: 0.82 ± 0.20
Yin et al. (2022) (99)	1,250 patients, multicenter study	3D MRI (T1c)	BMD	Internal and external validation	Detection	Recall: 93.2% False positive: 0.38
Park et al. (2021) (125)	282 patients, single-center study	3D MRI (BB, GRE)	3D U-Net	Internal validation	Detection and segmentation	Recall: 93.1% Dice score: 0.822
Yoo et al. (2022) (100)	65 patients, single-center study	3D MRI (T1c)	2D U-Net	Internal validation	Detection and segmentation	Recall: 97% Dice score: 75%
Liang et al. (2022) (95)	407 patients, multicenter study	3D MRI (T1c, T2-FLAIR)	3D DCNNs	Internal and external validation	Detection and segmentation	Dice score: 0.73 Recall: 0.91
Bouget et al. (2022) (126)	3,908 patients, multicenter study	3D MRI (T1c, FLAIR)	AGU-Net	Internal and external validation	Segmentation	Precision: 97.63 ± 00.77% Dice score: 87.73 ± 18.94% Recall: 97.46 ± 01.38%
Pflüger et al. (2022) (39)	338 patients, multicenter study	3D MRI (T1, T1c, FLAIR, T1 sub)	nnUNet	Internal and external validation	Detection	L-DICE Internal test set: 0.78 External test set: 0.79 L-Recall Internal test set: 0.81 External test set: 0.85
Ziyaee et al. (2022) (98)	1,051 patients, single-center study	3D MRI (T1c)	BM-Net + WB-Net	Internal validation	Detection and segmentation	Recall: 88.4% PPV: 90.1% Dice: 82.2%
Chartrand et al. (2022) (36)	530 patients, single-center study	3D MRI (T1c)	U-Net	Internal validation	Detection and segmentation	Recall: 90.9% Dice score: 0.73
Lee et al. (2023) (127)	2,149 patients, single-center study	3D MRI (T1c, T2)	Dual-pathway CNN	Internal validation	Segmentation	Dice score: 0.84
Li et al. (2023) (128)	649 patients, single-center study	3D MRI (T1, T1c, difference between T1 and T1c)	Two-stage deep learning model	Internal validation	Detection and segmentation	Recall: 90% Precision: 56% Dice score: 81%
Liew et al. (2023) (38)	677 patients, multicenter study	3D MRI (T1, T1c, T1-FLAIR)	NLMET	Internal and external validation	Detection	BrainMetShare Recall: 0.811 Local dataset Recall: 0.74 BrATS dataset Recall: 0.723
Guo et al. (2025) (23)	2,298 patients, multicenter study	3D MRI (T1c)	Extended nnUNet, ADS, ADL	Internal and external validation	Detection and segmentation	Recall: 0.904 Dice score: 0.758

### 2.3 DeepMedic and its variants

**Figure 4:**
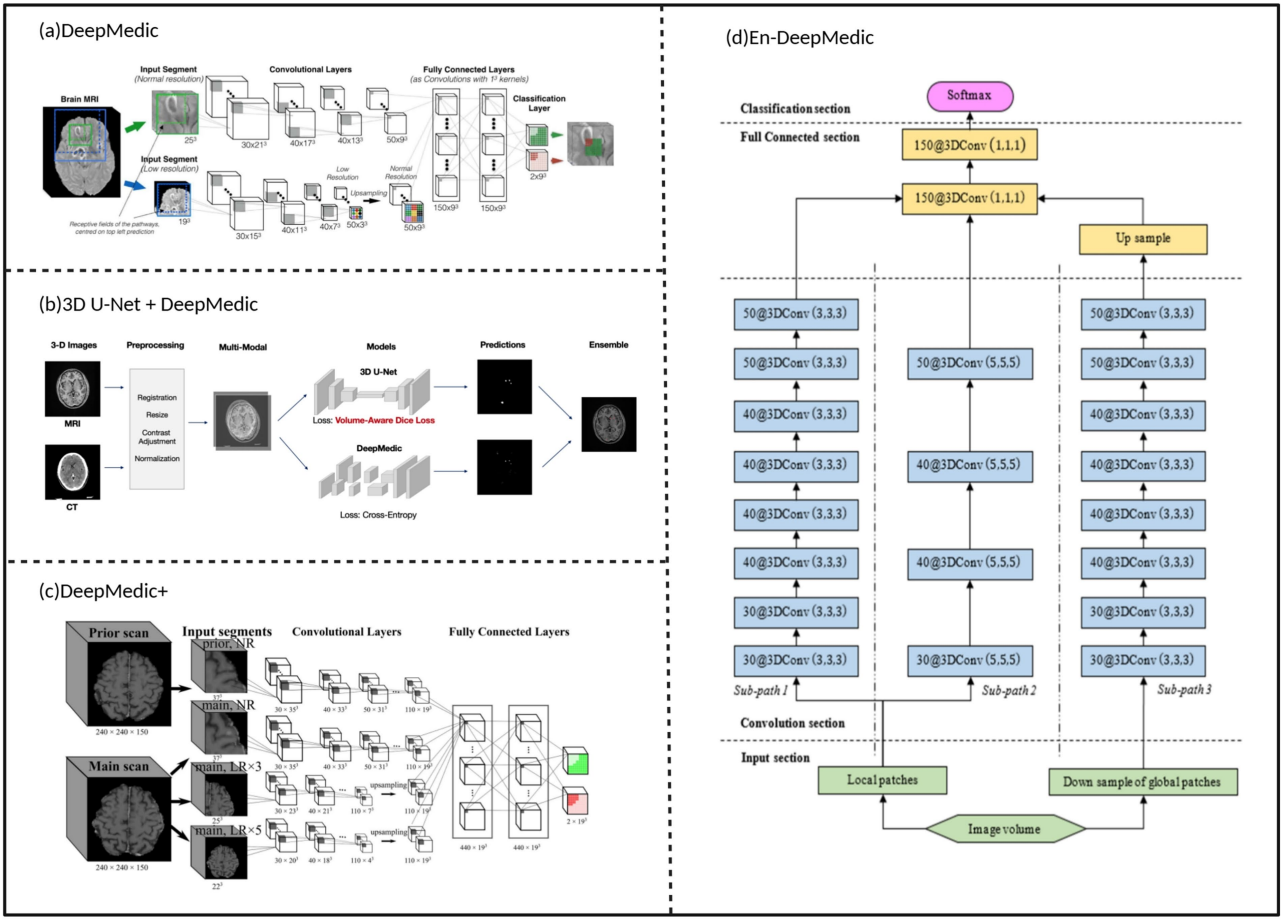
DeepMedic architecture for image segmentation of brain metastases. **(a)** Typical architecture of DeepMedic. **(b)** Commonly used structure of 3D U-Net integrated with DeepMedic. **(c)** Structure of DeepMedic+. **(d)**, Typical architecture of En-DeepMedic. Referenced and reproduced with permission from Kamnitsas et al. (44), Hu et al. (46), Huang et al. (11), and Liu et al. (45).

While U-Net technology continues to evolve, researchers are also exploring other architectural solutions specifically designed for 3D medical image segmentation (Figure 4). DeepMedic, as a CNN architecture specifically designed for 3D medical image segmentation tasks, was proposed by Kamnitsas et al. (44) in 2016. Unlike U-Net, which uses 2D CNNs and captures context and precise localization through contracting and symmetric expanding paths, DeepMedic employs a dual-path architecture that can simultaneously process input images at multiple scales, thereby better combining local and larger contextual information. This design enables DeepMedic to fully utilize volumetric data, capturing richer spatial information for more accurate segmentation of brain metastases. Additionally, DeepMedic employs a dense training scheme to effectively handle 3D medical scans and address class imbalance in the data, which contrasts with U-Net’s method of combining feature maps from contracting paths with expanding paths via skip connections to preserve high-resolution information. Another notable feature of DeepMedic is its use of a 3D fully connected conditional random field (CRF) for post-processing to remove false positives, further enhancing segmentation accuracy. Currently, DeepMedic has achieved state-of-the-art performance on multiple datasets, providing a new and effective tool for brain metastasis segmentation.

The emergence of DeepMedic attracted significant attention from researchers, leading to improvements and applications. Liu et al. (45) proposed En-DeepMedic, which adds extra sub-paths to capture more multi-scale features and utilizes GPU platforms to enhance computational efficiency, further improving segmentation accuracy, particularly for small lesions. Charron et al. (2) applied DeepMedic to segment brain metastases using multi-sequence MRI data (T1, T2, FLAIR), extending its application scope. Hu et al. (46) combined 3D U-Net with DeepMedic to process integrated MRI and CT images and proposed a volume-aware Dice loss to optimize segmentation by utilizing lesion size information, aiming to further improve small lesion detection. Jünger et al. (47) trained DeepMedic using data from heterogeneous scanners from different vendors and research centers, improving the model’s generalization and robustness, making it more applicable to clinical scenarios.

To further optimize DeepMedic’s performance, researchers have continually explored new methods and strategies. Huang et al. (11) introduced the volume-level sensitivity-specificity (VSS) loss function to balance sensitivity and specificity, addressing the difficulty DeepMedic had in reconciling these two aspects and further enhancing segmentation accuracy. Kikuchi et al. (48) combined DeepMedic with black and white blood images from the simultaneously acquired VISIBLE sequence, further improving detection sensitivity and reducing false positive rates, thus providing a more reliable basis for the accurate diagnosis of brain metastases.

Although DeepMedic and its improved versions have achieved good results in brain metastases segmentation (Table 3), existing technologies still have room for improvement in edge texture recognition of multiple lesions. To address this issue, multi-scale feature extraction and edge detection mechanisms can be integrated into the DeepMedic network architecture. Multi-scale feature extraction can enhance the model’s perception capability for lesions of different sizes, while edge detection can effectively capture edge texture information of lesions. The combination of these two approaches is expected to improve the accuracy of brain metastases image recognition.

**Table 3:** DeepMedic-based architecture for brain metastasis segmentation.

Author (year)	Dataset size and source	Imaging modality	Methodology	Model validation methods	Clinical outcomes predicted	Evaluation metrics
Kamnitsas et al. (2016) (44)	335 patients, multicenter study	3D MRI (FLAIR, T1, T1c, T2, DWI, PD)	DeepMedic	Internal and external validation	Segmentation	Dice score: 84.7% Precision: 85.0% Sensitivity: 87.6%
Liu et al. (2017) (45)	514 patients, multicenter study	3D MRI (T1c)	En-DeepMedic	Internal and external validation	Segmentation	BRATS dataset Tumor core Dice score: 0.75 ± 0.07 Enhanced tumor Dice score: 0.81 ± 0.04 AUC: 0.99 Clinical dataset Tumor core Dice score: 0.67 ± 0.03 AUC: 0.98 ± 0.01
Charron et al. (2018) (2)	182 patients, single-center study	3D MRI (T1 c, T2-FLAIR, T1)	DeepMedic	Internal validation	Detection and segmentation	Recall: 93% Dice score: 0.77
Hu et al. (2019) (46)	341 patients, single-center study	3D MRI, CT	3D U-Net + DeepMedic	Internal validation	Detection and segmentation	Dice score: 0.740 Precision: 0.779 Recall: 0.803
Jünger et al. (2021) (47)	98 patients, single-center study	3D MRI (T1, T2, T1 c, FLAIR)	3D DeepMedic	Internal validation	Detection and segmentation	Recall: 85.1% Dice score: 0.72 Precision: 68.7%
Park et al. (2022) (125)	176 patients, single-center study	3D MRI (T1c)	DeepMedic+	Internal validation	Detection and segmentation	DeepMedic + JVSS (*α* = 0.995) Recall: 0.932 Precision: 0.621 Dice score: 0.808
						DeepMedic + JVSS (*α* = 0.5) Recall: 0.842 Precision: 0.996 Dice score: 0.760
Kikuchi et al. (2022) (48)	84 patients, single-center study	3D MRI (VISIBLE)	DeepMedic	Internal validation	Detection	Recall: 91.7%

In terms of multi-scale feature extraction, inception modules or feature pyramid networks (FPN) can be introduced into the encoder part of DeepMedic. Inception modules effectively capture multi-scale information from images by using convolutional kernels of different sizes in parallel (such as 1 × 1, 3 × 3, 5 × 5, etc.), and have achieved good results in various image recognition tasks (49). FPN achieves effective fusion of features at different scales by constructing multi-level feature pyramids. For edge detection, an independent edge detection branch can be added after the output layer of DeepMedic, employing classical methods such as Sobel operators or Canny operators. The Sobel operator identifies edges by calculating the gradient of each pixel in the image in both horizontal and vertical directions, while the Canny operator is a more complex edge detection algorithm that can more accurately detect image edges and has the advantage of noise interference resistance through multi-level filtering and threshold processing (50). This improvement strategy can effectively extract edge information from segmentation results, thereby better identifying edge texture features of lesions and providing more reliable technical support for precise diagnosis and treatment of brain metastases.

Reviewing the development trajectory of CNN, U-Net, and DeepMedic architectures, the technological evolution logic of deep learning in the field of brain metastases analysis becomes clearly apparent. CNNs excel in local feature extraction but lack global contextual modeling capabilities, which directly resulted in high false positive rates in small lesion detection for early methods (such as the false positive rate of 0.05 per slice reported by Losch (31)). U-Net effectively addressed this limitation through its encoder-decoder structure and skip connection mechanisms. Its symmetric network design can both capture high-level semantic information and preserve low-level detailed features, thus generally outperforming early CNN methods in segmentation accuracy. DeepMedic adopts a dual-pathway design to simultaneously process inputs at different scales (44), possessing natural advantages when handling 3D volumetric data, although its computational complexity is relatively high.

From a performance perspective, U-Net-based adaptive frameworks demonstrate optimal application effectiveness, particularly the latest nnU-Net variants achieving over 90% sensitivity in detection tasks and Dice coefficients above 0.8 in segmentation tasks (13). However, this performance advantage comes at the cost of sacrificing interpretability, while the simple structure of CNNs makes feature visualization relatively straightforward, and DeepMedic’s dual-pathway design allows for separate analysis of contributions at different scales, providing certain advantages in interpretability. Regarding generalization ability, DeepMedic and nnU-Net perform relatively well, with the former showing good consistency across multi-center data (47) and the latter improving cross-dataset generalization ability through adaptive mechanisms (39).

Therefore, technology selection in clinical applications should be based on specific requirements: nnU-Net or improved 3D U-Net is recommended for high-precision scenarios, lightweight CNNs or 2.5D U-Net for real-time applications, DeepMedic or domain-adaptive U-Net should be prioritized for multi-center deployment, while scenarios requiring interpretability should employ CNNs combined with visualization techniques. Future research directions should focus on exploring effective integration of emerging architectures such as Transformers with existing frameworks, as well as designing composite loss functions optimized for small lesions, aiming to enhance model interpretability and generalization ability while maintaining high accuracy.

## 3 Brain metastases image classification tasks

### 3.1 Image-based differentiation between brain metastases and glioblastoma

**Figure 5:**
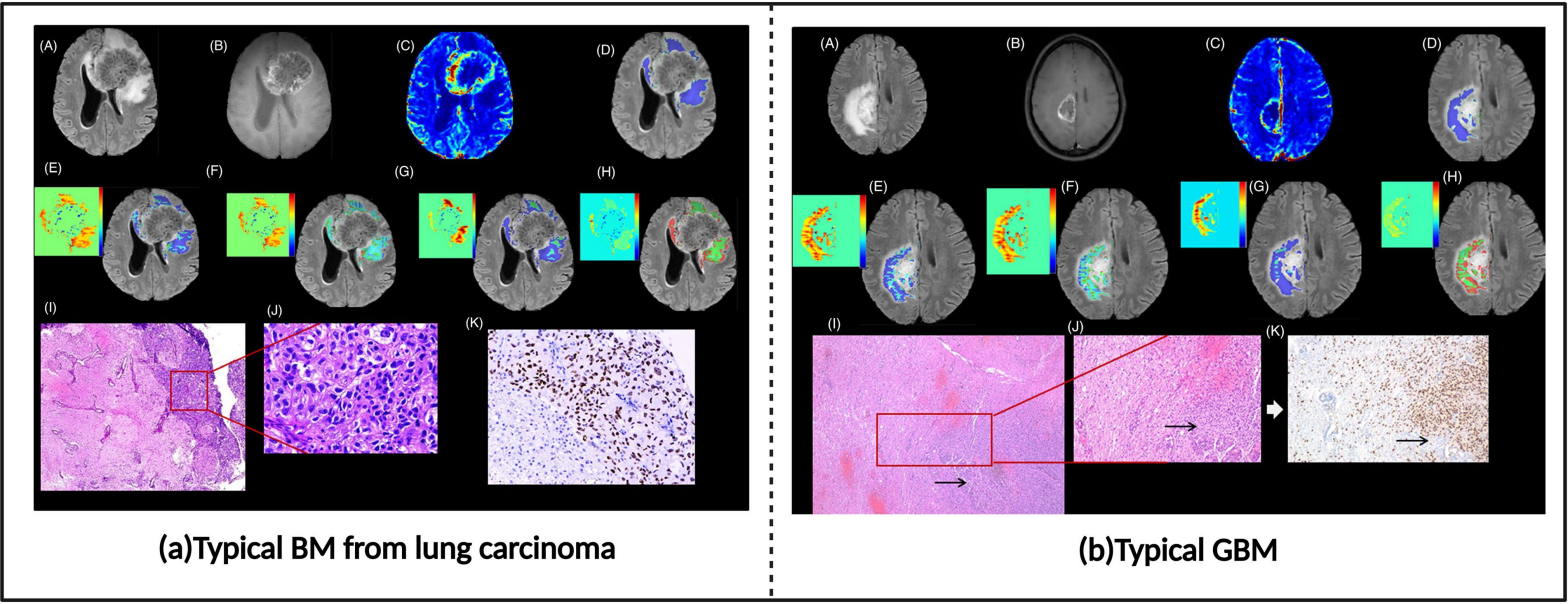
Brain metastases and glioblastoma images. **(a)** Typical BM from lung carcinoma. **(b)** Typical glioblastoma (GBM). Referenced and reproduced with permission from Parvaze et al. (58).

Brain metastases (BM) and glioblastoma (GBM) represent the most common malignant brain tumors in adults. For patients with pre-existing malignancies in other sites, accurate differentiation between brain metastases and primary glioblastoma when cerebral lesions appear holds significant clinical importance (51). Brain metastases demonstrate high similarity to glioblastoma multiforme on conventional MRI, with both potentially exhibiting rim enhancement with surrounding T2 hyperintensity, ring enhancement, and intratumoral necrosis (51, 52). These similar morphological presentations make accurate differentiation based solely on conventional imaging challenging (53). However, compared to glioblastoma multiforme, brain metastases typically feature more well-defined margins and a more spherical shape. Additionally, the peritumoral region of brain metastases primarily manifests as vasogenic edema, whereas glioblastoma multiforme peritumoral areas often show tumor cell infiltration with irregular shape and invasive growth characteristics (51, 52). Accurate differentiation based on these feature distinctions is crucial for treatment strategy formulation, as brain metastasis patients may receive systemic therapy targeting the primary tumor and local treatments such as SRS, while glioblastoma multiforme requires comprehensive treatment including maximal safe resection followed by molecular classification and concurrent chemoradiotherapy (51, 54). Evidently, accurate diagnosis not only avoids unnecessary invasive examinations and reduces patient risk but also improves diagnostic efficiency and provides a basis for timely treatment. In recent years, with the rapid development of imaging technologies and artificial intelligence, researchers have continuously explored new imaging methods and analytical techniques to improve the preoperative differential diagnostic accuracy between GBM and BM (Figure 5).

Radiomics, an emerging imaging analysis technique, provides powerful tools for the differential diagnosis of GBM and BM (Supplementary Table S1). By extracting a large number of quantitative features from medical images, such as first-order statistics, histogram features, and texture features (e.g., absolute gradient, gray-level co-occurrence matrix, gray-level run-length matrix, gray-level size zone matrix, and neighborhood gray-difference matrix), radiomics can effectively mine diagnostic information hidden in imaging data, thus improving the accuracy of distinguishing between GBM and BM.

Researchers such as Qian et al. (53), Artzi et al. (54), and Priya et al. (52) have extracted radiomic features and used various machine learning classifiers, including support vector machines (SVM) and random forests, to build differential diagnosis models for GBM and BM, achieving high diagnostic accuracy. Some researchers have begun to explore radiomics models based on multiparametric MRI to obtain more comprehensive tumor information. Liu et al. (55) extracted radiomic features from T2-weighted and contrast-enhanced T1-weighted images and built a tree-based pipeline optimization tool (TPOT) model to differentiate GBM from BM. The results showed that the model, incorporating both MRI sequences, achieved the best predictive performance. Bijari et al. (56) extracted hidden features from four 3D MRI sequences (T1, T2, T1c, FLAIR) and generated accurate features highly correlated with model accuracy. By using logistic regression combined with multidimensional discrete wavelet transformation, a multitask learning model was implemented to distinguish GBM from BM. Huang et al. (57) treated the 1,106 features extracted from each sequence (T1, T2, T1c) as three separate tasks, using a logistic loss function as a data fitting term to build a feature selection classification model for GBM and BM classification. Parvaze et al. (58) extracted 93 radiomic features from multiparametric MRI (FLAIR, T1c, T1) and used random forests to differentiate GBM from BM. Joo et al. (59) extracted radiomic features from T1, T2, T2 FLAIR, and T1c images and developed an integrated machine learning model based on LASSO feature selection and Adaboost, SVC classifiers for multiclass classification of glioblastoma, lymphoma, and metastases. Gao et al. (60) showed that extracting diffusion kurtosis imaging (DKI) parameters and conventional MRI sequence radiomic features, combined with various machine learning algorithms, could effectively differentiate GBM from SBM. The multi-DKI parameter model demonstrated the best diagnostic performance compared to single DKI parameter and conventional MRI models. These studies show that multitask learning strategies can effectively utilize complementary information between different MRI sequences, thus improving diagnostic efficiency and accuracy. Chen et al. (61) developed a diagnostic model combining texture features from the entire tumor area and the 10 mm tumor-brain interface area, using ANOVA1LR, KW1LR, RELIEF4NB, and RFE5NB algorithms to differentiate GBM from isolated brain metastasis (BM). In summary, radiomics provides an objective and accurate approach for the differential diagnosis of GBM and BM by extracting and analyzing multidimensional imaging features and using machine learning algorithms to construct predictive models, with promising clinical applications.

Convolutional neural networks (CNN), as an optimized deep learning technique, also show significant advantages in distinguishing GBM from BM (Supplementary Table S2). CNN models, with their unique structure, can automatically extract and learn multi-level features from imaging data that are difficult for traditional imaging analysis methods to extract and quantify (51, 62), such as tumor boundary clarity, features of internal necrotic areas, and infiltration of surrounding tissue. Then, through end-to-end training, the feature extraction and classification process is gradually optimized, effectively capturing subtle differences between GBM and BM and improving diagnostic accuracy. This end-to-end training mechanism allows CNNs to gradually learn abstract features from raw images, ranging from low-level features like edges and textures in shallow convolutional layers to more complex patterns like tumor shape, structure, and spatial distribution in deeper layers.

Bae et al. (51) and Shin et al. (63) respectively built differential diagnosis models for GBM and BM using deep neural networks (DNN) and ResNet-50, achieving diagnostic performance superior to that of junior neuroradiologists. This suggests that deep learning models can reach or even exceed human experts’ performance in some cases. To better utilize imaging information, researchers have developed classification models based on 3D CNNs. Chakrabarty et al. (62) developed a 3D CNN algorithm for classifying six common brain tumors, including GBM and BM, and achieved good classification results on T1-weighted MRI scans. The 3D CNN effectively captures the spatial information of tumors, improving diagnostic performance. In addition, multiparametric MRI is widely used in deep learning models. Yan et al. (64) used a 3D ResNet-18 algorithm and multiparametric MRI (DWI and conventional MRI) to construct a differential diagnosis model for GBM and BM, finding that the model combining DWI and conventional MRI had a higher AUC than single MRI sequence models, indicating that multimodal imaging data provide richer diagnostic information. Xiong et al. (65) used the GoogLeNet model and preoperative multiplanar T1-weighted enhanced (T1CE) MRI images to automatically differentiate high-grade gliomas (HGG) from solitary brain metastasis (SBM). The model achieved an average accuracy of 92.78% in distinguishing HGG from SBM, with over 90% accuracy even when distinguishing using only the tumor core or edema region. To further enhance clinical reliability, Park et al. (66) proposed a deep ensemble network based on DenseNet121, processing multiparametric MRI images to differentiate GBM and BM. This model not only provides accurate diagnostic results but also offers predictions of uncertainty and interpretability, enhancing clinicians’ trust in the model. In summary, deep learning methods, by automatically learning and analyzing complex imaging features, provide new and effective tools for the differential diagnosis of GBM and BM, advancing precision medicine.

Although the combination of imaging technology and AI has made significant progress in the differential diagnosis of GBM and BM, further research is needed to overcome existing challenges. Future studies should develop interpretable deep learning models, such as using heatmaps and Grad-CAM methods to explain model predictions, improving their clinical application value. Additionally, the development of automated tools, such as fully automated image segmentation and feature extraction tools, can enhance research efficiency and model robustness (55, 59, 60). In conclusion, future research needs breakthroughs in expanding sample sizes, integrating multimodal imaging data, exploring more detailed tumor sub-region analysis, combining clinical information, and enhancing model interpretability, to ultimately achieve accurate differential diagnosis of GBM and BM, providing better decision support tools for clinicians.

### 3.2 Classification of brain metastases sources

Accurate identification of brain metastases origin holds significant importance in clinical practice, as brain metastases from different primary sites exhibit marked differences in treatment responsiveness and prognosis. For example, brain metastases from small cell lung cancer (SCLC) and non-small cell lung cancer (NSCLC) are suitable for chemosensitivity therapy and surgery combined with targeted therapy, respectively (67), while brain metastases from breast cancer and melanoma may be more amenable to corresponding molecular targeted therapies or immunotherapies (68). These differences in treatment options directly impact patient survival benefits. However, in the absence of definitive primary lesion information, traditional tissue biopsy, although capable of determining the primary site, not only carries surgical risks and increases patient suffering but also proves intolerable for some patients due to factors such as poor physical condition or lesion location. Furthermore, when facing different pathological subtypes from the same organ, such as distinguishing between SCLC and NSCLC for refined classification, pathological biopsy alone often cannot provide sufficiently comprehensive information. Additional auxiliary methods such as immunohistochemical staining, molecular pathological detection, or genetic testing are typically required to clarify specific typing (67). Failure to promptly and accurately identify the origin leads to difficulties in treatment selection, affecting the optimal therapeutic window. Therefore, developing non-invasive imaging-based methods for brain metastases origin identification, using artificial intelligence technology to assist image analysis as an important complementary tool for clinical diagnosis, providing rapid and reliable auxiliary diagnostic information for clinical practice, holds significant value for optimizing treatment decisions and improving patient prognosis.

**Figure 6:**
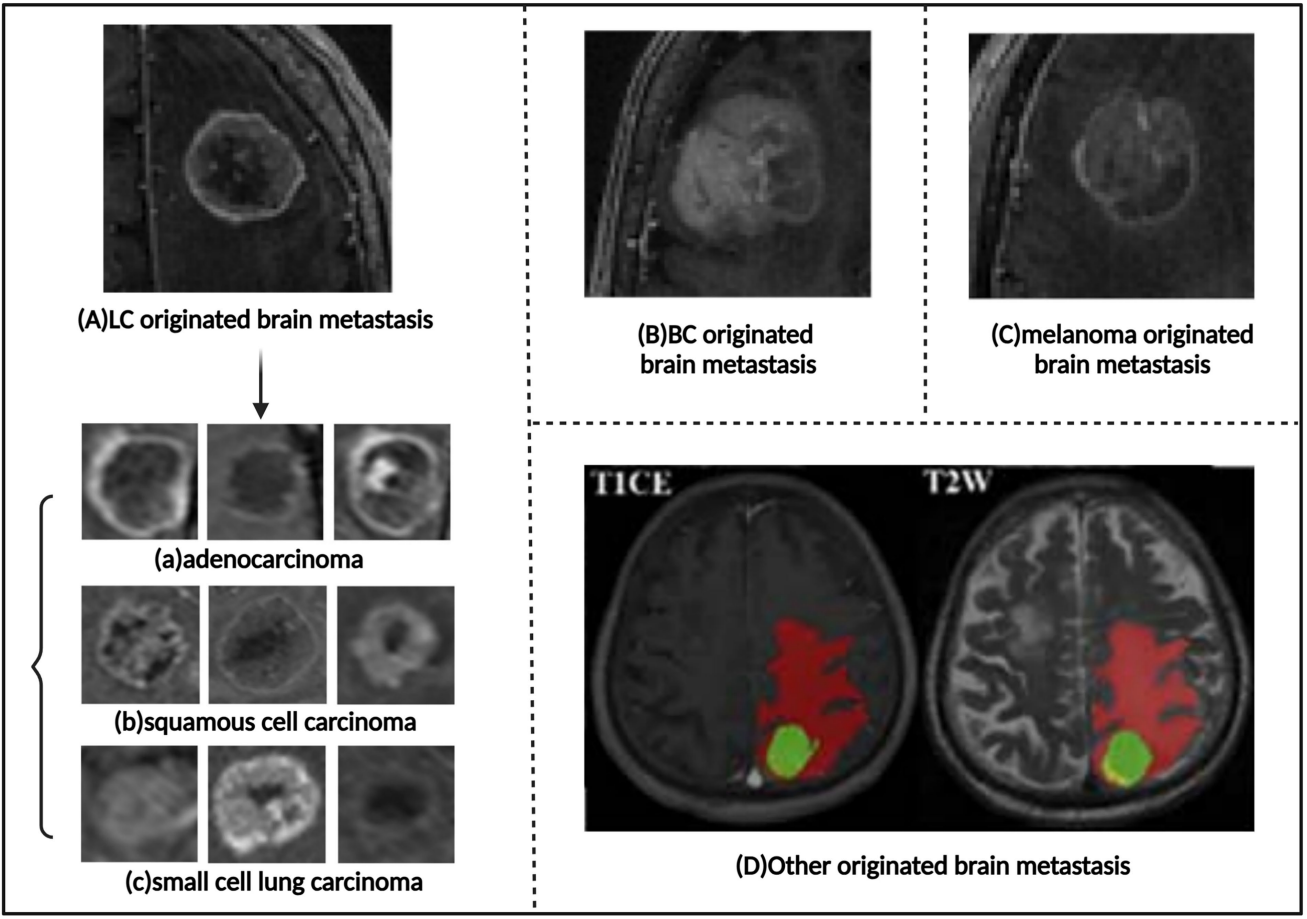
Images of brain metastases of different origins. **(A)** Lung carcinoma originated brain metastasis. The subtypes of brain metastases that arise from lung carcinoma include: **(a)** Adenocarcinoma. **(b)** Squamous cell carcinoma. **(c)** Small cell lung carcinoma. **(B)** Breast cancer originated brain metastasis. **(C)** Melanoma originated brain metastasis. **(D)**, Other originated brain metastasis. Referenced and reproduced with permission from Tulum (68), Ortiz-Ramón et al. (16), and Shi et al. (75).

However, traditional imaging diagnostic methods often struggle to accurately identify the source of brain metastases (Figure 6). Nonetheless, studies have shown that deep learning and machine learning methods can successfully classify the source of brain metastases (Supplementary Tables S3, S4). Image texture and radiomics analysis can extract subtle features from medical images that are difficult for the human eye to recognize, such as the uniformity, roughness, and directionality of the tumor’s internal gray-level distribution. These features are closely related to the tumor’s pathological characteristics, gene expression, and biological behavior, making them useful for distinguishing brain metastases originating from different primary tumors.

Classical machine learning methods have played an important role in the recognition of the primary source of brain metastases. Numerous studies have used machine learning methods to analyze texture features extracted from MRI or CT images in order to differentiate brain metastases originating from various primary tumors. Ortiz-Ramón et al. (16, 69, 70) conducted a series of studies exploring the impact of different texture features, classification models, and image modalities on brain metastasis classification. Early research (69) used 3D texture features and compared five classifiers: naive Bayes (NB), k-nearest neighbors (k-NN), multilayer perceptrons (MLP), random forests (RF), and linear kernel support vector machines (SVM). The study found that the NB classifier performed the best (AUC = 0.947 ± 0.067). Further research (70) focused on 2D texture features and used SVM and k-NN classifiers for evaluation. The results showed that the SVM classifier, combined with two gray-level co-occurrence matrix features, achieved a higher AUC (0.953 ± 0.061). In a subsequent study (16), they compared 2D and 3D texture features and found that 3D texture features were more advantageous in distinguishing brain metastases from different primary tumors. Using 3D texture features with 32 gray levels and a random forest classifier, they achieved an AUC of 0.873 ± 0.064. Béresová et al. (71) used texture analysis techniques [local binary pattern (LBP) and gray-level co-occurrence matrix (GLCM)] to extract image features and applied discriminant function analysis (DFA) to differentiate brain metastases from lung cancer and breast cancer. They compared texture features from contrast-enhanced T1-weighted images and LBP images and found that LBP image texture features were more effective in distinguishing lung cancer and breast cancer brain metastases, achieving an accuracy of 72.4%.

Kniep et al. (72) combined radiomics features with clinical data and used random forests to predict five different types of metastatic tumors, achieving AUC values ranging from 0.64 to 0.82. Zhang et al. (73) used radiomic features from brain CT images, combined with age and gender information, and applied binary logistic regression and SVM models to differentiate brain metastases from primary lung adenocarcinoma and squamous carcinoma, with AUC values of 0.828 and 0.769, respectively. Cao et al. (74) constructed and evaluated logistic regression and SVM models using selected radiomic features from individual CT, MRI, and combined images. The model showed the highest accuracy in differentiating brain metastases from lung cancer and breast cancer origins, with AUC values of 0.771 and 0.805, respectively. Tulum (68) combined traditional machine learning (SVM and MLP based on radiomics) and deep learning (EfficientNet-b0 and ResNet-50) to differentiate different subtypes of lung cancer brain metastases from MRI images. Although traditional machine learning methods performed well with small datasets, deep learning methods, through transfer learning, demonstrated higher classification performance on small datasets. Shi et al. (75) expanded the application range of radiomics by using LASSO regression to select multi-region radiomics features and then using logistic regression to differentiate brain metastases from lung adenocarcinoma and breast cancer origins. They also predicted epidermal growth factor receptor (EGFR) mutations and human epidermal growth factor receptor 2 (HER2) status, providing new insights for personalized treatment of brain metastasis patients. Mahmoodifar et al. (76) focused on the spatial distribution features of brain metastases. They used principal component analysis (PCA) to reduce the dimensionality of the spatial coordinates of brain metastases and combined age, target volume, and gender information with random forests, SVM, and TabNet deep learning models to differentiate brain metastases from five different primary cancer types. The SVM algorithm achieved an accuracy of 97%, and the TabNet model reached 96%.

These studies demonstrate that texture and radiomic features extracted from MRI or CT images, combined with appropriate machine learning models (68, 77), can effectively differentiate brain metastases from different primary tumors and predict relevant molecular marker statuses (75), providing new tools and strategies for the diagnosis, differential diagnosis, and personalized treatment of brain metastases. Compared to traditional machine learning methods, convolutional neural network-based deep learning models can automatically learn complex features in images without manual design or extraction of texture features, thus improving classification efficiency. For example, CNN models like EfficientNet (67, 68) and ResNet (68, 78–80) have achieved remarkable results in differentiating brain metastases from small cell lung cancer and non-small cell lung cancer, with accuracies reaching over 90%. Additionally, the application of 3D residual networks (3D-ResNet), combined with attention mechanisms, has further enhanced the model’s ability to capture key information, thus improving classification accuracy. For example, in a study (78), the use of a 3D-ResNet model for analyzing multi-sequence MRI data successfully increased the classification accuracy of small cell lung cancer versus non-small cell lung cancer brain metastasis from 85 to 92%.

### 3.3 Classification of radiation necrosis and tumor recurrence

**Figure 7:**
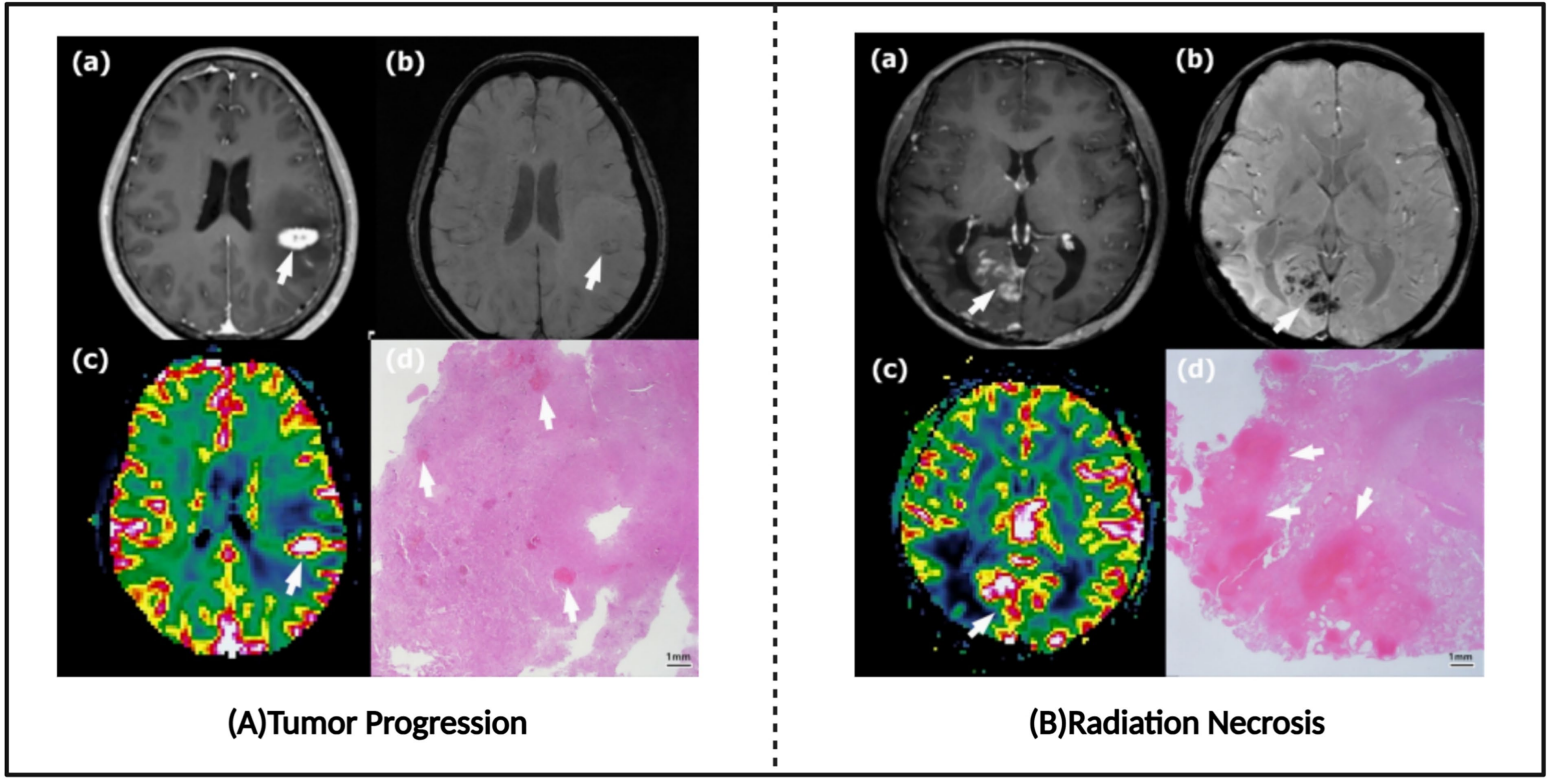
Tumor progression images and radio-necrosis images. **(A)** Typical tumor progression. **(B)** Typical radiation necrosis. Referenced and reproduced with permission from Kim et al. (87).

Radiation necrosis (RN) represents a significant late complication of SRS, with an incidence rate of 2.5–24%, predominantly occurring within 2 years post-treatment (81–83). When brain metastasis patients demonstrate new enhancing lesions on MRI after SRS treatment, differentiation between radiation necrosis and recurrent brain metastases becomes essential (Figure 7). Patients with radiation necrosis should avoid further radiotherapy to prevent exacerbation of necrosis, selecting non-invasive pharmacological treatment based on symptom severity or, when necessary, undergoing craniotomy to remove necrotic tissue. Conversely, tumor recurrence requires continued aggressive anti-tumor therapy, with options including repeated stereotactic radiosurgery or surgical resection. However, existing research indicates that conventional MRI alone typically cannot reliably distinguish between post-radiation radiation necrosis and recurrent tumors (84), presenting a challenge for clinical decision-making. Although biopsy with histopathological evaluation remains the gold standard for differential diagnosis, stereotactic biopsy may encounter sampling bias in mixed lesions containing both post-radiation radiation necrosis and recurrent tumors, making it difficult to obtain representative tissue samples (83). Furthermore, tissue biopsy not only carries inherent surgical risks, including complications such as hemorrhage, infection, and neurological function impairment, but also cannot be repeatedly performed as a routine monitoring method, significantly limiting its application value in dynamic assessment. Therefore, developing cost-effective non-invasive imaging diagnostic methods with high sensitivity and specificity holds significant clinical value for accurately differentiating between post-radiation radiation necrosis and recurrent tumors, as well as guiding individualized treatment decisions.

After brain tumor patients receive radiotherapy, new enhanced lesions often appear on magnetic resonance imaging (MRI), which could be either tumor recurrence or benign radiation necrosis. These two conditions often appear similar in imaging features (Figure 7), making differentiation a challenging task. Accurate differentiation is crucial for formulating subsequent treatment plans and improving patient prognosis. Traditional MRI sequences, such as T1-weighted imaging, T2-weighted imaging, and fluid-attenuated inversion recovery (FLAIR), form the basis for differential diagnosis. By observing the signal characteristics of lesions across different sequences, such as T1/T2 signal differences and lesion morphology, an initial judgment can be made regarding the nature of the lesion. However, these traditional MRI sequences often suffer from low sensitivity, making it difficult to reliably differentiate between tumor recurrence and radiation necrosis on their own (84). To improve diagnostic accuracy, various advanced artificial intelligence (AI) techniques have been introduced into clinical practice in recent years (Supplementary Table S5).

Larroza et al. (85) extracted 179 texture features, used recursive feature elimination with support vector machines (SVM) to select 10 important features, and then built a classification model with an SVM classifier. The results showed that the model achieved an area under the curve (AUC) of 0.94 ± 0.07 on the test set, demonstrating the potential of image texture-based analysis for distinguishing brain metastasis and radiation necrosis. Radiomics analysis has started to focus on the extraction and application of texture features in multiparametric MRI (such as T1c, T2, FLAIR, etc.) (86). For example, Tiwari et al. (86) utilized radiomic features extracted from multiparametric MRI and applied an SVM classifier to differentiate brain radiation necrosis and recurrent brain tumors, with FLAIR sequence achieving the highest AUC of 0.79. This suggests that combining multimodal imaging information can further improve diagnostic accuracy. Furthermore, Kim et al. (87) extracted radiomic features from magnetic susceptibility-weighted imaging and dynamic susceptibility contrast-enhanced perfusion imaging, and used logistic regression models to identify the best predictive factors for distinguishing recurrence and radiation necrosis. Their selected two predictive factors achieved 71.9% sensitivity, 100% specificity, and 82.3% accuracy. Yoon et al. (88) used volumetric weighted voxel-based multiparametric clustering to analyze parameters such as ADC, nCBV, and IAUC, achieving an AUC of 0.942–0.946. Zhang et al. (89) extracted 285 radiomic features from T1, T1 enhanced, T2, and FLAIR sequences, and used the RUSBoost ensemble classifier to construct a model with a prediction accuracy of 73.2%. Peng et al. (90) employed 3D texture analysis and a random forest classifier, achieving higher classification accuracy (AUC >0.9). Their study found that 3D texture features were more suitable for differentiating brain metastases from lung cancer compared to breast cancer and melanoma, and random forests performed better with fewer features. This study also provided a potential non-invasive diagnostic tool for brain metastasis patients of unknown primary origin. Chen et al. (91) extracted multiparametric radiomics features and used random forest algorithms to construct a classification model, achieving an AUC of 0.77 in the training cohort and 0.71 in the validation cohort. Salari et al. (92) extracted radiomic features from MR contrast-enhanced T1-weighted images and used random forest algorithms, achieving an AUC of 0.910 ± 0.047. Basree et al. (17) analyzed radiomic features from T1 enhanced, T2, and FLAIR sequences and used logistic regression models for prediction, achieving an AUC of 0.76 ± 0.13. Zhao et al. (93) extracted image features from 3D MRI scans, collected 7 clinical and 7 genomic features, and fused them using position encoding in a heavy ball neural ordinary differential equations (HBNODE) model to predict radiation necrosis or recurrence after SRS for BM, achieving an ROC AUC of 0.88 ± 0.04, sensitivity of 0.79 ± 0.02, specificity of 0.89 ± 0.01, and accuracy of 0.84 ± 0.01.

Although deep learning has made significant progress in medical image analysis, it has not yet been widely applied to directly differentiate radiation necrosis from recurrent tumors after radiotherapy. Current research primarily focuses on extracting radiomic features from images and constructing classifiers using classical machine learning methods. There is currently a lack of studies using deep learning methods, such as convolutional neural networks (CNNs), to distinguish between radiation necrosis and tumor recurrence after radiotherapy. This may be closely related to the dependence of deep learning models on large annotated datasets. Since cases of radiation necrosis and recurrence are relatively few, there is a shortage of training samples, which is one of the major factors limiting the performance of deep learning models. Furthermore, the problem of data imbalance exacerbates this challenge. Radiation necrosis cases are often far fewer than recurrence cases, leading to model bias towards the majority class during training, which weakens the model’s ability to recognize the minority class. This imbalance is particularly pronounced in tasks that require high precision to distinguish between two similar pathological states, significantly affecting the model’s classification performance. At the same time, acquiring high-quality annotations is also challenging. Annotating medical images requires in-depth expertise and relies on annotators’ extensive clinical experience. However, subjective differences between different doctors and inconsistencies in annotations by the same doctor at different time points can introduce noise into the data, adversely affecting the model’s training outcomes. These factors together limit the widespread application of deep learning in distinguishing radiation necrosis from recurrent tumors.

However, deep learning algorithms have the ability to automatically learn complex features from medical images, eliminating the need for manual feature extraction. In practical applications, deep learning models shorten diagnostic cycles and improve efficiency through fully automated processes. Additionally, deep learning models exhibit strong adaptability and robustness, being able to handle imaging data from different modalities and resolutions. This demonstrates the vast potential for deep learning in distinguishing radiation necrosis from recurrent tumors. Despite challenges such as limited data availability, data distribution imbalance, and difficulty in acquiring high-quality annotations, targeted and effective solutions are gradually emerging through further research and practical exploration. Regarding sample size expansion, data augmentation techniques (20, 94, 95) can generate new samples with similar distributions to the original data by performing transformations such as rotation, scaling, and cropping, effectively expanding the training dataset. To address the data imbalance issue, resampling techniques such as random oversampling, undersampling, and the SMOTE (synthetic minority over-sampling technique) algorithm (96) can adjust the sample proportion of different categories in the dataset, enabling the model to focus more on the minority class samples during training and improving its recognition ability for the minority class. Additionally, to solve the high-quality annotation issue, establishing standardized annotation processes and multi-expert consensus mechanisms is key. By setting detailed annotation guidelines and conducting cross-validation and annotation review with multiple experienced medical experts, the subjective differences and inconsistencies during annotation can be effectively minimized, thereby improving the quality and reliability of annotated data.

## 4 Challenges and future directions

In brain metastasis research, the application of machine learning has made significant progress, but there are still challenges in tasks such as detection, segmentation and classification, including issues such as small sample sizes, insufficient model generalization ability, and multimodal data integration. To address these challenges, researchers have actively explored various solutions. For instance, to overcome data limitations, techniques such as data augmentation (20, 94, 95), dense overlapping stitching (95), and transfer learning (67, 68) have been widely used. To improve model generalization ability, researchers have focused on domain generalization (38, 94), multi-center dataset training (97), and adaptive network architectures (13, 39, 98). Methods such as multi-channel input and feature fusion (99) have been used to integrate complementary information from multimodal MRI images. For specific tasks, researchers have also developed corresponding strategies. For example, in brain metastasis segmentation, methods such as asymmetric structures (21), multi-scale feature fusion (99), improved loss functions (36), and overlapping patch techniques (100) have been used to improve the sensitivity of small lesion detection. In the differentiation between GBM and BM, brain metastasis and radiation necrosis, researchers have not only focused on integrating multimodal imaging data (51, 54, 55, 60, 63, 84, 87, 101), but have also explored more detailed tumor sub-region analysis (14, 54, 58, 60, 61, 65) and integration of clinical information (57, 59, 102) to improve diagnostic accuracy.

However, current research still has several limitations (Supplementary Table S6). For example, although CT images play a key role in the early screening of brain metastasis, most current studies focus on MRI images, neglecting the potential applications of CT images in brain metastasis segmentation and classification tasks. Additionally, most of the existing studies have small sample sizes and lack multi-center validation, which limits the model’s generalization ability and clinical application value. Furthermore, the interpretability of deep learning models still needs improvement, and enhancing the transparency and trustworthiness of models will help integrate them more effectively into clinical workflows.

### 4.1 The gap between CT and MRI in brain metastasis image analysis

Deep learning has made significant progress in brain metastasis MRI image analysis, but incorporating CT images into the analysis pipeline holds important clinical significance and research value. First, CT examinations are more widespread and economical, especially in developing countries and primary healthcare settings, making CT a more accessible diagnostic tool. It is also more patient-friendly for individuals who are immobile or unable to tolerate long MRI scans. Additionally, CT images serve as the standard imaging basis for radiotherapy planning. Integrating CT images into brain metastasis segmentation and classification tasks can better assist in delineating radiotherapy target areas and dose calculation, improving the precision and safety of radiotherapy.

Although CT images are less commonly used in brain metastasis image segmentation and classification, some studies have explored this. In brain metastasis image segmentation, Wang et al. (103) constructed an improved U-Net architecture with a position attention module (PAM) to automatically segment the gross tumor volume (GTV) from CT simulation images of brain metastasis patients. This model demonstrated excellent performance in external independent validation sets, though its generalization ability needs further validation. Wang et al. (104) further innovated by combining GAN, Mask R-CNN, and CRF optimization to construct a deep learning model for automatic segmentation of GTV in brain metastasis from CT simulation images. The model demonstrated good generalization ability on both internal and external validation datasets, providing an effective technical approach for brain metastasis image segmentation. However, despite advances in CT image segmentation technology in brain metastasis diagnosis, its performance still lags behind MRI and requires further optimization.

In brain metastasis image classification, existing research has attempted to use CT radiomics features and deep learning models. For example, Li et al. (105) used CT radiomics features from lung cancer patients to predict brain metastasis, achieving good diagnostic performance (AUC = 0.81). Zhang et al. (106) constructed a stacked ensemble model for classifying tumor volume (GTV), brainstem, and normal brain tissue in brain metastasis CT images, outperforming individual base models (AUC = 0.928, 0.932, and 0.942, respectively). Gong et al. (107) proposed a deep learning model combined with CT radiomics features to predict the risk of brain metastasis in non-small cell lung cancer patients within 3 years. Their ensemble learning model showed good predictive efficacy on both training and validation sets (AUC between 0.85–0.91). While CT images have been applied in brain metastasis classification, the lower image clarity and resolution compared to MRI make it more challenging to distinguish brain metastasis from normal tissue in CT images. As a result, models trained on CT images typically perform worse in feature extraction, classification accuracy, and generalization ability compared to models trained on MRI images, limiting the depth and breadth of research in brain metastasis CT image classification. However, globally, especially in developing countries and primary healthcare settings, CT remains an important diagnostic tool due to its higher prevalence, lower cost, ease of access, and greater convenience for patients unable to tolerate long MRI scans. Therefore, CT continues to play a crucial role in brain metastasis diagnosis and related research, prompting researchers to address the limitations of CT images and improve the performance of models based on CT images.

In future research, considering the differences between CT and MRI in imaging principles and clinical application advantages, and recognizing that they cannot replace each other, it may be valuable to combine both modalities to more comprehensively assess brain metastasis characteristics. Exploring deep learning models based on fused CT and MRI images, such as developing automatic brain metastasis segmentation models or classification models, could improve segmentation and classification accuracy, leading to more precise treatment planning.

### 4.2 The conflict between AI model generalization and patient privacy protection

Deep learning models show great potential in the diagnosis and treatment of brain metastasis, offering innovative solutions and breakthrough possibilities in this field. However, a key factor limiting the widespread clinical application of deep learning models is the lack of sufficient external validation. This issue leads to insufficient model generalization, making it difficult for the models to adapt to the complex and dynamic clinical scenarios.

Insufficient model generalization is a common issue in medical imaging research. In brain metastasis segmentation research, some studies lack external validation on independent test sets, rely only on single-center data, or lack multi-center data for external validation. Some studies also use multimodal MRI data and cascaded networks, but with small training datasets from single institutions, making it difficult to adapt to different hospital scanning technologies and hardware differences, limiting the generalization ability of the models and potentially leading to performance degradation in real-world applications. Similar issues arise in the differential diagnosis of GBM and BM, identification of brain metastasis sources, and the differentiation of radiation necrosis and tumor recurrence post-radiotherapy. Many studies lack external dataset validation, making it difficult to ensure the models’ effectiveness in diverse environments. Some studies also suffer from small sample sizes and focus only on limited tumor types, resulting in poor model generalization ability. Furthermore, some studies also face the combined challenges of small sample sizes, lack of external independent validation, and pathology diagnosis verification, reducing the reliability of the results and severely limiting the model’s generalization ability, making it difficult to apply in broader clinical settings. The limitations in model generalization performance are not challenges unique to brain metastasis segmentation tasks. The medical image analysis field has addressed similar issues through establishing large-scale clinical validation datasets via multi-institutional collaborations, while simultaneously utilizing these standardized datasets to provide unified accuracy assessment metrics (such as sensitivity, specificity, Dice coefficient, etc.), thereby enabling direct performance comparison and objective evaluation between different algorithms. The primary brain tumor segmentation validation framework represented by the brain tumor segmentation (BRATS) challenge has thoroughly validated the effectiveness of this multi-center data-driven approach in enhancing algorithm clinical translation capabilities, providing a successful paradigm that can be referenced for brain metastasis image analysis tasks (18).

To promote the clinical application of machine learning technologies in brain metastasis segmentation and classification, validation is needed on larger, more diverse clinical datasets to assess the models’ reliability and effectiveness. However, constructing large-scale, diverse brain metastasis datasets also presents challenges, especially in head and neck imaging data. Unlike imaging data from other parts of the body, head and neck images contain a significant amount of facial information, which is highly identifiable and reconstructible, and direct public use could lead to patient privacy breaches. Therefore, when building public datasets, strict anonymization processes, such as face blurring or de-identification, are necessary to ensure patient privacy. This is one of the reasons why head and neck tumor imaging data in public databases like TCIA are difficult to share openly.

However, while strict anonymization can address privacy concerns to some extent, the variability of data from different hospitals introduces new challenges. Differences in scanners, imaging parameters, and patient populations at different hospitals can make generalization ability even more crucial for clinical applications. To improve model generalization, domain adaptation/domain generalization techniques (108) can be used to overcome distribution differences between datasets, for example, by learning common features across domains or regularizing the model to enhance its robustness to different data distributions. Additionally, federated learning techniques (109) can be used to train models on multi-center data while protecting patient privacy. For example, Jiménez-Sánchez et al. (110) proposed a federated learning method combining curriculum learning and unsupervised domain adaptation, which achieved significant results in classification performance (AUC 0.79, PR-AUC 0.82, far surpassing traditional methods) and domain adaptation in breast cancer classification. Feng et al. (111) built a robust federated learning model (RFLM) using multi-center preoperative CT imaging data of gastric cancer patients, outperforming clinical models and other federated learning algorithms in predicting post-surgery recurrence risk. Federated learning methods, including horizontal, vertical, and federated transfer learning, can be selected based on specific situations. Horizontal federated learning is suitable for cases where participants have similar features but different samples, such as brain metastasis patient data from different hospitals. Vertical federated learning is suitable when participants have the same samples but different features, such as data from different departments within the same hospital. Federated transfer learning is applicable when participants have both different samples and features. Additionally, data heterogeneity, communication efficiency, and privacy concerns must be considered. Techniques such as differential privacy and homomorphic encryption can further enhance privacy protection in federated learning.

For brain metastasis diagnosis and treatment, federated learning can be used to integrate data from multiple medical institutions, thereby training deep learning models with better generalization ability. For example, a federated learning network involving multiple hospitals can be built to collaboratively train a brain metastasis segmentation model using each hospital’s imaging data, without sharing raw patient image data, effectively protecting patient privacy.

However, despite federated learning demonstrating enormous potential at the technical level, it still faces complex administrative coordination and policy regulation challenges in practical applications, which perhaps explains why most current large-scale medical imaging databases tend to adopt the traditional model of multi-source anonymized data integration. In the future, federated learning technology needs continuous optimization in algorithm robustness, privacy protection mechanisms, and heterogeneous data processing capabilities to better adapt to the practical requirements of complex medical image analysis tasks such as brain metastases.

### 4.3 AI model interpretability and clinical trust challenges

Although deep learning models have achieved excellent performance in brain metastases detection, segmentation, and classification tasks, their “black box” characteristics severely constrain clinical translation applications. AI interpretability challenges in brain metastases diagnosis are particularly prominent, as clinicians need to understand how AI distinguishes microscopic lesions smaller than 3 mm from vascular artifacts, the basis for determining lesion boundaries, and the prioritization logic in cases with multiple lesions. This lack of decision transparency directly affects physicians’ trust in AI systems, becoming a critical barrier to clinical adoption.

Current interpretability methods exhibit obvious limitations in brain metastases applications. Although Adnan et al. (112) employed Grad-CAM technology to visualize model attention regions, with their NASNet large model achieving 92.98% accuracy while providing clear localization, the interpretation granularity is coarse and difficult to meet precise diagnostic requirements. Chen et al. (113) used SHAP methods to analyze 10 mm brain-tumor interface features, with their logistic regression model achieving an AUC of 0.808 and quantifying feature contribution values, but the computational complexity of high-dimensional image processing limits real-time applications. The integrated gradients method by Sayres et al. (114) improved physician sensitivity from 79.4 to 88.7%, yet simultaneously exposed the double-edged effect of interpretability—potentially increasing misdiagnosis risk for patients without lesions. These studies indicate that existing interpretability techniques lack optimization design specifically for brain metastases tasks.

The impact of interpretability deficiency has transcended the technical level, becoming a significant barrier for AI systems to obtain regulatory approval, hospital procurement decisions, and clinical workflow integration. In clinical practice, radiologists’ acceptance of AI recommendations highly depends on their understanding of decision logic, particularly when handling complex cases or formulating treatment plans. Current brain metastases AI research generally treats interpretability as an additional feature rather than a core requirement, leading to a disconnect between technological development and clinical needs.

Future brain metastases AI systems should construct multi-level, personalized interpretability frameworks. At the technical level, comprehensive solutions integrating LIME local interpretation, Grad-CAM global visualization, and uncertainty estimation are needed, incorporating brain anatomical prior knowledge and radiomics semantic features. At the clinical level, stratified interpretation interfaces should be designed for physicians with different experience levels, providing detailed educational explanations for residents and key feature summaries for senior physicians. At the system level, standardized metrics for interpretability evaluation and multi-center validation mechanisms need to be established to ensure clinical effectiveness and safety of interpretation methods. More importantly, deep integration of interpretable AI with clinical decision support systems should be promoted, constructing a fully transparent diagnostic and treatment system from image analysis to treatment recommendations, truly achieving collaborative development between AI technology and clinical practice.

### 4.4 Lack of clinical practice translation and reader studies

Although deep learning models have shown significant potential in the diagnosis and treatment of brain metastases, their clinical translation still faces numerous challenges. For instance, while the U-Net architecture and its improved models have achieved small-scale clinical applications in brain metastasis image segmentation, they still face significant limitations in terms of precision for small lesion detection and generalization ability, particularly when adapting to different scanning devices and MRI sequences (20, 21). These technical limitations severely restrict large-scale clinical translation and application. Similarly, in brain metastasis image classification tasks, machine learning-based models for the differentiation of glioblastoma (GBM) and brain metastasis (BM) show high diagnostic accuracy in internal validation, but their clinical application remains significantly limited. The main reason is that these models are often based on single-center retrospective studies, lacking multi-center external validation, leading to concerns about their reliability across different medical institutions and patient populations (51, 53, 54). Furthermore, while studies combining radiomics and machine learning have made some progress in differentiating brain metastasis subtypes, the lack of standardized feature selection and model optimization processes has resulted in poor reproducibility and consistency between studies, severely affecting the clinical deployment value of these models (69, 70, 96).

To better serve clinical practice, AI research should establish a standardized validation process system comprising three levels: technical validation, clinical validation, and implementation validation. The technical validation phase should adopt multi-dimensional assessment metrics including Dice coefficient, sensitivity, specificity, and Hausdorff distance, while introducing clinical relevance evaluation. Clinical validation requires designing prospective multi-center randomized controlled reading studies (110), objectively evaluating the impact of AI systems on diagnostic accuracy, reading time, and clinical decision-making by randomly assigning radiologists of different experience levels to AI-assisted groups and control groups. The key is to establish unified MRI scanning parameter standardization protocols, including technical specifications such as contrast agent injection timing for T1c sequences and slice thickness settings, as well as image quality control standards, ensuring consistency and comparability of multi-center research data.

To achieve genuine clinical application of AI systems, key issues such as technical integration and regulatory compliance must be addressed. In PACS system integration, interface design based on DICOM standards should be adopted, developing structured report formats compliant with DICOM-SR standards to achieve seamless storage and retrieval of AI analysis results in PACS (115). Through asynchronous processing modes and automatic triggering mechanisms, the system should be capable of automatically processing newly uploaded brain MRI examinations without affecting normal hospital workflow. In regulatory compliance, quality management systems meeting the requirements of regulatory agencies such as FDA and NMPA must be established, including complete software lifecycle management, risk control measures, and continuous performance monitoring mechanisms. AI system performance dashboards should be established to monitor key indicators such as processing time and accuracy in real-time, automatically alerting and initiating emergency responses when performance deviates from preset thresholds. Additionally, improving clinical physicians’ acceptance is equally critical. Training programs should be designed for medical personnel at different levels, helping clinicians understand the advantages and limitations of AI systems through case analysis and practical exercises, and establishing user feedback collection mechanisms to continuously optimize system functionality.

In reader studies, existing research exhibits obvious inadequacies. Although studies indicate that deep learning-assisted systems (BMSS) can significantly improve the accuracy and efficiency of brain metastases delineation, particularly with more pronounced effects for less experienced residents (116), these studies are mostly limited to single-center small-sample data, resulting in lack of generalizability. Compared to fields such as breast cancer detection and lung cancer detection (117, 118), reader studies for AI in brain metastases segmentation and classification tasks are relatively lacking. Current research focuses more on technical-level algorithm optimization and performance improvement, with less involvement in evaluating radiologists’ performance when using AI tools in actual clinical practice. Future research should pay more attention to radiologists’ performance when using AI-assisted systems, particularly the differences among physicians of different experience levels when using AI tools. It is recommended to design multi-center, multi-level reader studies to evaluate AI tool performance in different clinical scenarios and explore their potential value in training young physicians, better guiding the practical application of AI in clinical settings.

## 5 Conclusion

Artificial intelligence technologies, including classical machine learning and deep learning, have shown enormous potential in the diagnosis and treatment of brain metastases. From precise tumor segmentation to complex classification tasks, AI technologies provide new tools to improve diagnostic accuracy and efficiency. Deep learning models such as U-Net and DeepMedic have achieved significant results in brain metastasis detection and segmentation tasks, while machine learning and deep learning methods have also been successfully applied to differentiate brain metastases from glioblastoma, identify primary sources of brain metastases, and distinguish radiation necrosis from tumor recurrence post-radiotherapy. Although AI has made promising progress in brain metastasis image analysis, further research is still needed to overcome existing challenges, such as improving model interpretability and generalization ability, building large-scale high-quality clinical datasets, developing user-friendly software tools, and conducting rigorous clinical trials. With continued technological advancements and deeper clinical application, AI technologies are expected to make greater contributions to the precision diagnosis and prognosis improvement of brain metastases.
