# Supplementary material for: Artificial intelligence in the task of segmentation and classification of brain metastases images: current challenges and future opportunities
Source: Front Neurol. 2025 Sep 23;16:1581422. doi: 10.3389/fneur.2025.1581422 (PMC12500441; doi:10.3389/fneur.2025.1581422)
Supplement: Supplementary file 1 [file Table_1.docx]

**Table S1:** Classical Machine Learning Methods for Brain Metastasis (BM) and Glioblastoma (GBM) Classification

| Author (year) | Dataset size and source | Imaging modality | Methodology | Model validation methods | Clinical outcomes predicted | Evaluation metrics |
| --- | --- | --- | --- | --- | --- | --- |
| Qian et al. (2019) [53] | 412 patients, single-center study | 3D MRI (T1, T2, CE) | SVM | Internal and external validation | Classification | Recall: 0.80 Specificity: 0.87 Accuracy: 0.83 |
| Artzi et al. (2019) [54] | 439 patients, single-center study | 3D MRI (T1c) | SVM | Internal validation | Classification | AUC: 0.96 Specificity: 0.85 Accuracy: 0.85 Recall: 0.86 |
| Priya et al. (2021) [52] | 120 patients, single-center study | 3D MRI (T1, T2, T1c, ADC, FLAIR) | LASSO | Internal validation | Classification | AUC: 0.953 |
| Liu et al. (2022) [55] | 935 patients, multicenter study | 3D MRI (T2, T1c) | TPOT | Internal and external validation | Classification | AUC: 0.867 |
| Bijari et al.  (2022) [56] | 91 patients, single-center study | 3D MRI (T1, T2, T1c, FLAIR) | LR | Internal validation | Classification | Accuracy: 0.98 AUC: 0.99 |
| Huang et al. (2022) [57] | 187 patients, multicenter study | 3D MRI (T1, T2, T1c) | SVM | Internal and external validation | Classification | AUC: 0.992 Accuracy: 0.920 Precision: 0.969 Recall: 0.871 |
| Parvaze et al.  (2023) [58] | 83 patients, single-center study | 3D MRI (T1c, FLAIR), 2D MRI (T2c), SWI, DWI | RF | Internal validation | Classification | AUC: 0.84 Accuracy: 76.66% |
| Joo et al. (2023) [59] | 707 patients, multicenter study | 3D MRI (T1, T2, T2 FLAIR, T1c) | LASSO+Adaboost+SVC (ensemble learning) | Internal and external validation | Classification | Accuracy: 76.3% AUC: 0.878 |
| Gao et al. (2024) [60] | 110 patients, single-center study | 3D MRI (DKI, T2, T2 - dark - fluid, DWI, T1c) | SVM | Internal validation | Classification | AUC: 0.958 Accuracy: 0.909 Sensitivity: 0.917 Specificity: 0.900 |
| Chen et al. (2025) [61] | 187 patients, single-center study | 2D MRI (T1c) | LR | Internal validation | Classification | AUC: 0.893 Accuracy: 0.857 |

**Table S2:** Deep Learning Methods for Brain Metastasis (BM) and Glioblastoma (GBM) Classification

| Author (year) | Dataset size and source | Imaging modality | Methodology | Model validation methods | Clinical outcomes predicted | Evaluation metrics |
| --- | --- | --- | --- | --- | --- | --- |
| Bae et al. (2020) [51] | 248 patients, multicenter study | 3D MRI (T2, T1c) | DNN | Internal and external validation | Classification | AUC: 0.956 Recall: 90.6% Specificity: 88.0% Accuracy: 89.0% |
| Shin et al. (2021) [63] | 741 patients, multicenter study | 2D MRI (T2, T1c) | ResNet-50 | Internal and external validation | Classification | AUC: 0.835 Recall: 88.9% Accuracy: 85.9% Precision: 0.907 |
| Chakrabarty et al. (2021) [62] | 2105 patients, multicenter study | 3D MRI (T1c) | 3D CNN | Internal and external validation | Classification | Recall: 98% Specificity: 99% AUC: 1.00 |
| Yan et al. (2023) [64] | 234 patients, multicenter study | 3D MRI (T1, T2, T2-FLAIR, T1c, DWI) | 3D ResNet-18 | Internal and external validation | Classification | AUC: 0.886 Accuracy: 80.7% |
| Xiong et al.  (2024) [65] | 209 patients, multicenter study | 2D MRI (T1c) | GoogLeNet | Internal and external validation | Classification | Accuracy: 89.66% Recall: 90.91% Specificity: 83.33% |
| Park et al. (2024) [66] | 612 patients, multicenter study | 2D MRI (T2, T1c) | Deep ensembles | Internal and external validation | Classification | AUC: 0.83 Accuracy: 76.2% Recall: 54.8% Specificity: 85.2% |

**Table S3:** Classical Machine Learning Methods for Brain Metastasis (BM) Origin Identification

| Author (year) | Dataset size and source | Imaging modality | Methodology | Model validation methods | Clinical outcomes predicted | Evaluation metrics |
| --- | --- | --- | --- | --- | --- | --- |
| Ortiz-Ramón et al. (2017) [69] | 30 patients, single-center study | 2D MRI (T1) | NB | Internal validation | Classification | AUC: 0.947±0.067 |
| Ortiz-Ramón et al. (2017) [70] | 29 patients, single-center study | 2D MRI (T1) | SVM | Internal validation | Classification | AUC: 0.953±0.061 |
| Béresová et al. (2018) [71] | 58 patients, single-center study | 2D MRI (T1) | LM (Linear Model) | Internal validation | Classification | AUC: 0.683 |
| Ortiz-Ramón et al. (2018) [16] | 38 patients, single-center study | 3D MRI (T1) | RF | Internal validation | Classification | AUC: 0.873±0.064 |
| Kniep et al. (2019) [72] | 189 patients, single-center study | 3D MRI (T1, T1c, FLAIR) | RF | Internal validation | Classification | Melanoma:  AUC: 0.82 Recall: 74% Specificity: 76% Non–small Cell Lung Cancer:  AUC: 0.64 Recall: 58% Specificity: 62% |
| Zhang et al. (2021) [73] | 144 patients, single-center study | CECT | BLR | Internal validation | Classification | AUC: 0.828 Recall: 55.8% Specificity: 92.3% Accuracy: 74.1% |
| Cao et al. (2022) [74] | 78 patients, single-center study | CT, MRI (T1) | SVM | Internal validation | Classification | AUC: 0.805 Recall: 74.2% Specificity: 87.0% |
| Tulum et al. (2023) [68] | 74 patients, single-center study | 3D MRI (T2, FLAIR, T1c) | ResNet-50 | Internal validation | Classification | Recall: 94.29% Specificity: 94.08% |
| Shi et al. (2023) [75] | 160 patients, single-center study | 3D MRI (T2, T1c) | LASSO | Internal validation | Classification | AUC:  RS-LA: 0.778 RS-BC: 0.843 RS-EGFR: 0.729 RS-HER2: 0.784 |

**Table S4:** Deep Learning Methods for Brain Metastasis (BM) Origin Identification

| Author (year) | Dataset size and source | Imaging modality | Methodology | Model validation methods | Clinical outcomes predicted | Evaluation metrics |
| --- | --- | --- | --- | --- | --- | --- |
| Grossman et al. (2021) [67] | 69 patients, single-center study | 2D MRI (T1, T1c, T2, FLAIR) | EfficientNet | Internal validation | Classification | NSCLC: Precision: 0.89±0.10 Recall: 0.88±0.07 Accuracy: 0.88±0.05 |
|  |  |  |  |  |  | SCLC: Precision: 0.83±0.10 Recall: 0.88±0.11 |
| Tulum et al. (2023) [68] | 74 patients, single-center study | 2D MRI (T1c) | EfficientNet+ResNet-50 | Internal validation | Classification | AUC: 97.4% Specificity: 94.08% Accuracy: 94.14% |
| Jiao et al. (2023) [78] | 214 patients, multicenter study | 3D MRI (T1, T2, T2FLAIR, DWI, T1c) | 3D-ResNet | Internal and external validation | Classification | AUC:  LC vs NLC: 0.684±0.089 NSCLC vs SCLC: 0.800±0.100 |
| Li et al. (2024) [79] | 246 patients, multicenter study | 3D MRI (T1, T2, T2-FLAIR, DWI, T1c) | ARFN | Internal and external validation | Classification | AUC:  NSCLC vs SCLC: 0.751 AD vs SCC: 0.738 |
| Zhu et al. (2024) [80] | 250 patients, multicenter study | 2.5D MRI (T1C, FLAIR) | ResNet101 | Internal validation | Classification | AUC: 0.868 Accuracy: 0.836 |

**Table S5:** Classical Machine Learning Methods for Distinguishing Recurrent Brain Metastasis from Radiation Necrosis

| Author (year) | Dataset size and source | Imaging modality | Methodology | Model validation methods | Clinical outcomes predicted | Evaluation metrics |
| --- | --- | --- | --- | --- | --- | --- |
| Larroza et al. (2015) [85] | 73 patients, single-center study | 3D MRI (T1c) | SVM | Internal validation | Classification | Dataset 1:  AUC: 0.94±0.07 Dataset 2:  AUC: 0.93±0.02 |
| Tiwari et al. (2016) [86] | 58 patients, multicenter study | 2D MRI (T1c, T2, FLAIR) | SVM | Internal and external validation | Classification | AUC: 0.79 Accuracy: 80% |
| Kim et al. (2017) [87] | 51 patients, single-center study | 3D MRI (SWMRI, DSC PWI) | LR | Internal validation | Classification | Recall: 71.9% Specificity: 100% Accuracy: 82.3% |
| Yoon et al. (2017) [88] | 75 patients, single-center study | 2D MRI | MP | Internal validation | Classification | AUC: 0.942-0.946 Recall: 95.2%-97.6% |
| Zhang et al. (2018) [89] | 87 patients, single-center study | 3D MRI (T1, T1c, T2, FLAIR) | RUSBoost | Internal validation | Classification | AUC: 0.73 Accuracy: 73.2% |
| Peng et al. (2018) [90] | 66 patients, single-center study | 2D MRI (T1c, T2, FLAIR) | LR | Internal validation | Classification | Recall: 65.38% Specificity: 86.67% AUC: 0.81 |
| Chen et al. (2021) [91] | 109 patients, multicenter study | 3D MRI (T1c, T2-FLAIR) | RF | Internal and external validation | Classification | Recall: 52% Specificity: 90% AUC: 0.71 |
| Salari et al. (2023) [92] | 86 patients, multicenter study | 3D MRI (T1c) | RF | Internal validation | Classification | AUC: 0.910 ± 0.047 Accuracy: 0.8 ± 0.071 Recall: 0.796 ± 0.055 Specificity: 0.922 ± 0.059 |
| Basree et al. (2024) [17] | 55 patients, single-center study | 2D MRI (T1c, T2, FLAIR) | LR | Internal validation | Classification | AUC: 76.2% Specificity: 75.5% Recall: 62.3% |
| Zhao et al. (2025) [93] | 62 patients, single-center study | 3D MRI (T1c) | HBNODE | Internal validation | Classification | AUC: 0.88±0.04 Recall: 0.79±0.02 Specificity: 0.89±0.01 |

**Table S6**: Summary of key challenges and limitations in current brain metastases AI research

| Limitations | Core Manifestations | Solution Strategies | Key Technical Approaches | Expected Outcomes |
| --- | --- | --- | --- | --- |
| Data Scale Constraints | • Small sample sizes •Difficult data acquisition •High annotation costs | Data augmentation and transfer learning strategies | •Data augmentation techniques [33,37,41] •Dense overlapping patching [41] •Transfer learning [101,105] •Generative adversarial network data augmentation | Improve model training effectiveness and alleviate small-sample learning difficulties |
| Insufficient Generalization Capability | •Lack of multi-center validation •Single-institution data limitations •Large inter-device variations | Domain generalization and multi-center collaboration | •Domain generalization algorithms [38,94] •Multi-center dataset training [97] •Adaptive network architectures [13,39,98] •Federated learning technologies | Enhance model adaptability and stability across different clinical environments |
| Multi-modal Integration Challenges | •Insufficient utilization of multi-modal MRI information •Difficulties in inter-modal feature fusion •Inadequate complementary information mining | Multi-modal feature fusion optimization | •Multi-channel input design [99] •Feature fusion algorithms [99] •Attention mechanism integration •Cross-modal learning strategies | Fully utilize multi-modal information to improve diagnostic accuracy |
| Imaging Modality Imbalance | •Research overly focused on MRI images •Underutilized potential of CT images | CT-MRI collaborative analysis framework | •CT image preprocessing optimization •CT-MRI registration techniques •Cross-modal transfer learning •Multi-modal decision fusion | Establish a more comprehensive brain metastases imaging analysis system |
| Insufficient Task-Specific Optimization | •Limited precision in small lesion detection | Task-oriented algorithm optimization | •Asymmetric network structures [21] •Multi-scale feature fusion [99] •Improved loss functions [36] • Overlapping patch techniques [100] | Significantly improve small lesion detection and segmentation accuracy |
| Clinical Feature Integration Deficiency | •Separation of imaging and clinical information •Lack of multi-dimensional comprehensive analysis | Multi-dimensional information fusion strategies | •Multi-modal imaging data integration [51, 54, 55, 60, 63, 84, 87, 101] • Tumor subregion analysis [14, 54, 58, 60, 61, 65] • Clinical information integration [57,59,102] • Radiomics feature extraction | Construct more accurate diagnostic and prognostic assessment models |
| Model Interpretability Deficiency | •Deep learning "black box" characteristics •Lack of decision transparency •Insufficient clinical trust •Difficulty integrating into clinical workflows | Explainable AI technology development | • Attention mechanism visualization • Feature importance analysis • Decision pathway tracing • Clinical knowledge-guided explanations | Enhance model transparency and credibility to promote clinical applications |
